# Metabolic resilience governs sex-specific pain recovery during hormonal aging: a multi-omics study of neuropathy in mice

**DOI:** 10.3389/fpain.2025.1655712

**Published:** 2025-10-13

**Authors:** Sara Marinelli, Claudia Rossi, Luisa Pieroni, Giacomo Giacovazzo, Valentina Vacca, Federica De Angelis, Ilaria Cicalini, Valentina Mastrorilli, Chiara Parisi, Zuleyha Nihan Yurtsever, Domenico Ciavardelli, Roberto Coccurello

**Affiliations:** ^1^ National Research Council of Italy (CNR), Institute of Biochemistry and Cell Biology, Monterotondo, Italy; ^2^ Center for Advanced Studies and Technology (CAST), “G. d’Annunzio” University of Chieti-Pescara, Chieti, Italy; ^3^ Departmental Faculty of Medicine and Surgery, UniCamillus—Saint Camillus International University of Health Sciences, Rome, Italy; ^4^ IRCCS San Camillo Hospital, Venice, Italy; ^5^ The Department of Veterinary Medicine, European Center for Brain Research/Institute for Research and Health Care (IRCCS) Santa Lucia Foundation, Rome, Italy; ^6^ School of Veterinary Medicine, University of Teramo (UniTE), Teramo, Italy; ^7^ Department of Science, “G. d'Annunzio” University of Chieti-Pescara, Chieti, Italy; ^8^ School of Medicine and Surgery, “Kore” University of Enna, Enna, Italy; ^9^ National Research Council of Italy (CNR), Institute for Complex System (ISC), Rome, Italy

**Keywords:** neuropathic pain, allodynia, sex differences, aging, adipose tissue, energy metabolism, acylcarnitines, proteomic

## Abstract

**Introduction:**

Biological aging and sex interact to shape systemic metabolism, yet their role in chronic pain resolution remains unexplored. We hypothesized that metabolic resilience—the ability to flexibly switch fuel sources and maintain energy homeostasis—rules successful recovery from nerve injury in a sex-dependent manner during aging.

**Methods:**

In 12-month-old male and female mice, corresponding to the perimenopausal phase in females and the onset of hormonal decline in both sexes, we induced sciatic nerve chronic constriction injury and performed multi-omics profiling during Wallerian degeneration, a phase known to trigger long-term neurobiological remodeling.

**Results:**

Aging females exhibited early activation of fatty acid oxidation, increased resting energy expenditure, upregulation of mitochondrial redox enzymes and circulating progesterone and corticosterone. Proteomic and metabolomic analysis revealed pentose phosphate pathway enrichment and gluconeogenesis, supporting redox balance and metabolic flexibility. Conversely, males displayed persistent glycolytic reliance, long-chain acylcarnitine accumulation, suppression of adiponectin and PPARγ, indicating metabolic inflexibility. Longitudinal behavioral analysis revealed that aging females recovered earlier and more fully than aging males, reversing the pattern previously shown in our adult mouse study, where females developed persistent pain and males recovered rapidly.

**Discussion:**

These patterns highlight a non-linear, sex-specific interaction between biological aging and injury response, where hormonal decline reprograms the metabolic trajectory and reshapes pain outcomes. Metabolic resilience governs sex-specific recovery following nerve injury by directing early systemic adaptations that precede and predict long-term pain trajectories. These results define mechanistically anchored, sex- and age-specific biomarkers, and propose preclinical targets for timely, personalized interventions in age-associated neuropathic pain.

## Introduction

Peripheral neuropathy (PN) is a highly prevalent condition in older adults, affecting up to 30% of individuals in certain populations (1). Its incidence increases with age and is disproportionately higher in peri- and postmenopausal women, likely due to the loss of estrogen-mediated neuroprotection and the resulting impairment in neural and immune homeostasis (2, 3). Clinically, PN involves sensory, motor, and autonomic dysfunctions that significantly impair quality of life. Mounting evidence indicates that age-related metabolic and inflammatory changes, coupled with altered body composition—such as increased central adiposity and sarcopenia—contribute to PN development (1, 4, 5). In this context, biological sex and aging act as modulators of systemic energy homeostasis, potentially influencing the trajectory of neuropathic pain (NeP) and its resolution (6).

We and others have previously shown that adipose tissue (AT) is not only an energy storage depot but also a dynamic endocrine organ, modulating both inflammation and nerve regeneration through the secretion of adipokines such as leptin and adiponectin (3, 7). In postmenopausal women, estrogen loss is associated with increased PN prevalence, which has been linked to impaired immune regulation and enhanced oxidative stress (8, 9). Estrogens support Schwann cell (SC) activity and protect peripheral nerves by preserving redox balance and glial function (8, 9). In adult female mice, peripheral nerve injury disrupts metabolic homeostasis, leading to early alterations in lipid metabolism, reduced energy expenditure, and dysregulated steroid secretion from AT, all of which contribute to impaired glycemic and insulin responses (3).

In contrast, male mice display a distinct metabolic response to nerve injury, characterized by enhanced glycolytic flux, lower energy expenditure, and altered fatty acid handling. Notably, AT in males remains functionally responsive, supporting mitochondrial adaptation, oxidative stress compensation, and neuro-regenerative signaling through adiponectin and PPARγ pathways (9–12). The reduced regenerative activity of SCs in females further delays axonal repair, revealing a complex interplay between sex hormones, glial responses, and metabolic plasticity.

Emerging data also emphasize the importance of AT innervation in maintaining systemic metabolic health. PN involving subcutaneous AT (scAT) disrupts nerve-adipose crosstalk, impairing substrate mobilization and endocrine signaling. Both myelinated and unmyelinated scAT fibers exhibit high plasticity, which is lost in pathological contexts such as neuropathy (13, 14). Dysfunctional SCs in scAT contribute to demyelinating phenotypes, underscoring their dual role in nerve maintenance and injury response (14, 15).

Although it is well recognized that aging alters peripheral nerve physiology (4, 16, 17), the mechanistic drivers of NeP onset, persistence, and resolution—particularly in the context of age and sex—remain poorly defined. Prior studies have proposed that nerve injury acts as a metabolic switch, triggering energy-intensive repair programs that must be tightly regulated to ensure functional recovery (3, 18–20). In this framework, metabolic inflexibility may serve as an early determinant of pain chronification, particularly in aging organisms where the ability to adapt energy substrates is compromised.

Here, we hypothesize that biological sex modulates the metabolic trajectory of nerve injury response in aging, defining distinct pain outcomes. We further propose that early metabolic rewiring—especially within AT—governs resilience or vulnerability to chronic pain, offering a window for biomarker-based stratification and sex-specific therapeutic targeting.

To test this, we employed a mouse model of sciatic nerve injury in 12-month-old male and female mice, representing the perimenopausal and early androgen-decline stage, and integrated behavioral, calorimetric, imaging, molecular, and multi-omics analyses. Our approach aimed to define mechanistically anchored, sex-dependent metabolic pathways involved in the early response to nerve injury and to identify predictive markers of long-term pain trajectories. By elucidating these processes, this work supports the development of personalized interventions to prevent or reverse chronic NeP in aging individuals.

## Methods

### Murine models of chronic constriction injury-induced neuropathic pain

**Figure 1:**
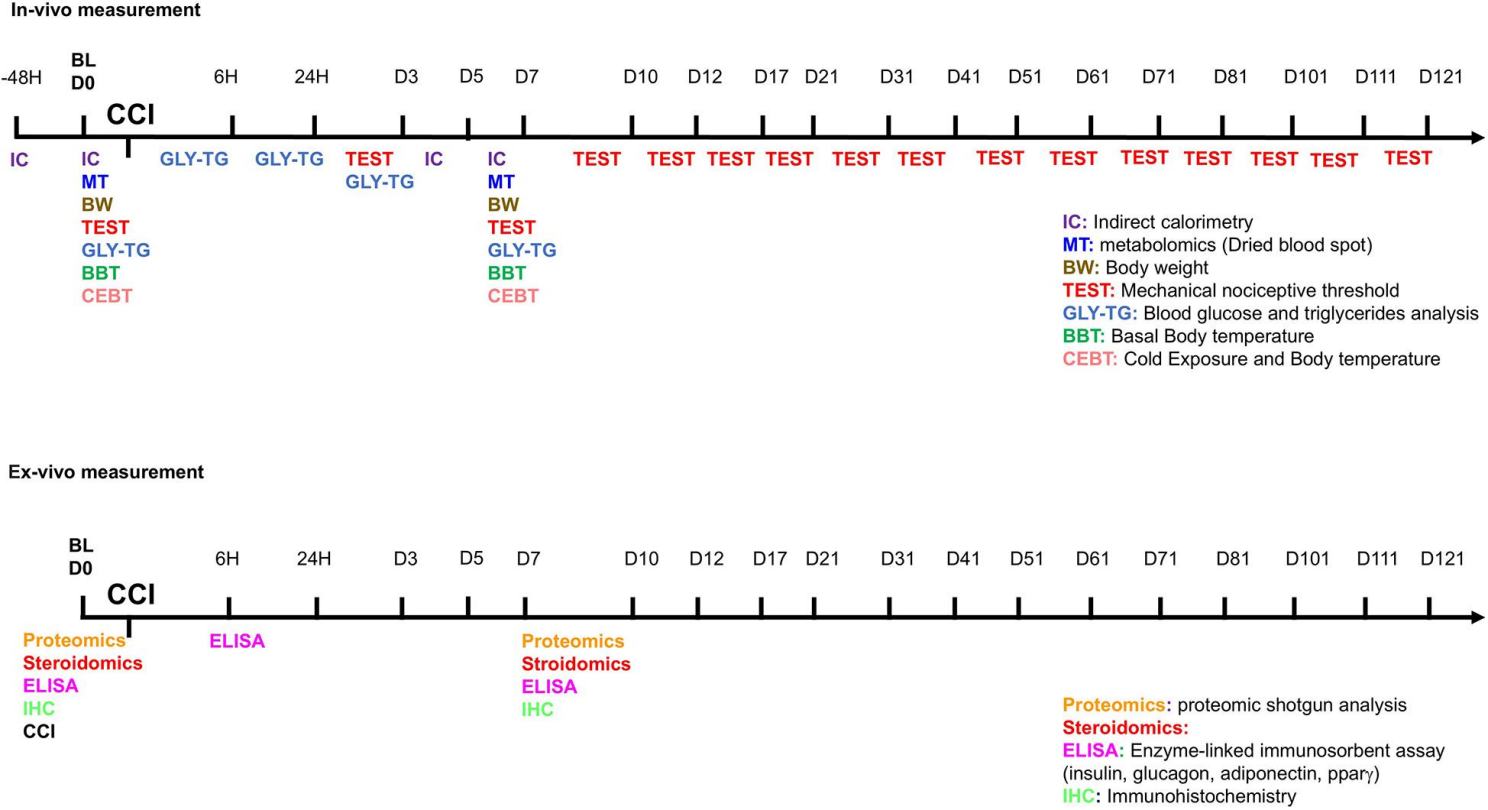
Timeline of experimental procedures. On the line, the hour (6 h- 24 h- 48 h) or the day of test (D0 to D121) before (baseline—BL) or after CCI are reported. In multicolor acronyms are indicated *in vivo* (top panel) or ex-vivo (bottom panel) experiments.

Male and female CD1(ICR)CD1 mice, approximately 12 months old, were obtained from Charles River Labs (Como, Italy) or the European Mouse Mutant Archive—EMMA Infrafrontier (Monterotondo RM, Italy) for this study. The animals were housed under standard conditions in transparent plastic cages, in groups of four, with sawdust bedding, following a 12 h light/dark cycle (07:00 AM–07:00 PM). Food and water were provided *ad libitum*. After behavioral testing, female estrous cycle stages were determined using vaginal smears. Since no significant differences were observed in behavioral responses across different estrous phases, all female mice were included in the same experimental group regardless of hormonal status. All procedures strictly adhered to European and Italian national regulations governing the use of animals in research (Legislative Decree No. 26 of 04/03/2014, implementing European Communities Council Directive 10/63/EU) and were approved by the Italian Ministry of Health (authorization numbers 32/2014PR and 164/2024PR). To induce NeP, the Chronic Constriction Injury (CCI) model was employed, adapted for mice from the original method described by Bennett and Xie (21) Surgery was performed under anesthesia using a 1:1 mixture of Rompun (Bayer, 20 mg/ml; 0.5 ml/kg) and Zoletil (100 mg/ml; 0.5 ml/kg). A longitudinal skin incision (∼1.5 cm) was made to expose the mid-portion of the right sciatic nerve. Three loosely tied ligatures (7–0 chromic gut, Ethicon, Rome, Italy) were placed around the nerve to induce partial constriction. The incision was then closed using 4–0 silk sutures. From this point forward, the injured right hindpaw will be referred to as the “ipsilateral paw,” while the uninjured left hindpaw will be termed the “contralateral paw.” Mice underwent behavioral assessments (*in vivo* experiments) or were sacrificed for tissue collection (ex vivo experiments) before CCI surgery to obtain baseline reference values. The experimental timeline is detailed in Figure 1. The study included male and female CD1 mice, evaluated before and at various time points after CCI surgery in *in vivo* experiments. The first cohort of mice underwent a series of assessments, arranged from the least to the most stressful to minimize experimental interference: allodynia evaluation, glycemia and triglyceride (TG) measurements, body temperature recording, and cold exposure followed by body temperature measurement. The second cohort was designated for indirect calorimetry experiments. For *ex vivo* analyses, each time point represented a separate group of male or female CD1 mice, as animals were sacrificed for tissue collection. The number of animals (N) in each group is specified in the figure legends, corresponding to the respective experimental conditions and statistical analyses.

### Assessment of mechanical allodynia (dynamic plantar aesthesiometer test)

Neuropathy onset was evaluated by assessing the sensitivity of both ipsilateral and contralateral hindpaws to normally non-painful mechanical stimuli at various time points, ranging from postoperative day 3 (D3) up to day 121 (D121). To measure nerve injury-induced mechanical allodynia, we employed the Dynamic Plantar Aesthesiometer (Ugo Basile, Model 37400), a device that progressively applies mechanical force over time. The force was directed to the plantar surface of the mouse hindpaw, and the nociceptive threshold was determined as the amount of force (in grams) at which the animal withdrew its paw. On each testing day, the withdrawal response of both ipsilateral and contralateral hindpaws was recorded across three consecutive trials, ensuring at least 10 s between each measurement. The final withdrawal threshold was calculated as the average of these three trials. Behavioral testing was conducted in both male and female mice.

### Measurement of glycemia and triglycerides

Blood glucose and triglyceride levels were assessed using the Multicare Test Strips system (Biochemical Systems International). Measurements were obtained through tail vein sampling in naïve animals (baseline, BL) and at multiple time points following chronic constriction injury (CCI), specifically at 6 h, 24 h, day 3 (D3), and day 7 (D7) (3). These assessments were conducted in both male and female mice (18).

### Enzyme-linked immunosorbent assay (ELISA) for insulin, glucagon, PPARγ, adiponectin, and leptin

Serum concentrations of insulin and glucagon were determined using ELISA kits (RayBio® Mouse Insulin ELISA Kit; RayBiotech Inc., Norcross, GA, USA, and Quantikine® ELISA Glucagon Immunoassay; R&D Systems), following previously established protocols (10). Leptin levels in serum and AT were quantified using the Mouse Leptin (LEP) ELISA Kit (BT LAB) according to the manufacturer's instructions, and absorbance values were obtained using the Varioskan Lux Instrument (ThermoFisher). Blood samples were collected at 6 h and 7 days post-CCI (*n* = 3 per time point) from both male and female mice. White adipose tissue (WAT) was dissected from the subcutaneous abdominal (inguinal) adipose panniculus. PPARγ and adiponectin levels in WAT lysates were measured using ELISA kits from MyBiosource Inc. (San Diego, USA) and Quantikine® ELISA Immunoassay (R&D Systems), respectively. Tissue samples were collected from both male and female mice (*n* = 3 per time point) at baseline and seven days post-CCI. All incubation and washing steps were performed according to the manufacturers' instructions, followed by the addition of a substrate solution. The intensity of the colorimetric reaction was directly proportional to the concentration of insulin and glucagon in serum, as well as PPARγ and adiponectin in AT. Absorbance was recorded at 450 nm, with wavelength correction at 570 nm for insulin, glucagon, and adiponectin detection.

### Immunohistochemical analysis

The sciatic nerve from mice in each experimental group was collected for immunofluorescence (IF) analysis. Animals were euthanized using a sub-lethal dose of a Rompun (Bayer) and Zoletil (Virbac) mixture, followed by perfusion with saline and 4% paraformaldehyde in phosphate-buffered saline (PBS, pH 7.4). The sciatic nerve was then extracted and immersed in 4% paraformaldehyde in PBS for 48 h. Subsequently, the tissue was cryoprotected in a 30% (w/v) sucrose solution in PBS before being stored at −80 °C. Cryostat-generated sections (20 µm) were mounted directly onto glass slides for further processing. IF analysis was conducted at baseline (BL) and seven days post-CCI (D7) in both male and female mice (12 months—12 M). For double IF staining, tissue sections were incubated overnight with primary antibodies targeting S100β (Schwann cell marker; mouse monoclonal, 1:100, Sigma-Aldrich: S2532) and IRS1 (Insulin receptor substrate 1; rabbit polyclonal, 1:100, Abcam) or CD11b (complement receptor 3/cluster of differentiation 11b, macrophages marker; rat anti-mouse, 1:100, Millipore: MCA711) and myelin protein 0 (P0—chicken polyclonal, 1:200, AB9352; Millipore). Both antibodies were diluted in 0.3% Triton-X100 (Sigma-Aldrich) for optimal penetration.

Following three washes in PBS, sciatic nerve sections were incubated at room temperature for 2 h with fluorescently labeled secondary antibodies: Alexa Fluor 488-conjugated donkey anti-mouse (1:100, Jackson ImmunoResearch) and Rhodamine-conjugated anti-rabbit (1:100, Jackson ImmunoResearch 1110251) or fluorescein-conjugated rat anti-mouse (FITC, 1:100, Jackson ImmunoResearch) or rhodamine-conjugated donkey anti-chicken DyLight 549 (DYL, 1:100, Jackson Immuno Research). After two additional PBS washes, sections were counterstained with the DNA-binding fluorochrome bisBenzimide (Hoechst, 1:1,000, Sigma-Aldrich) for 10 min. To attain specificity and eliminate nonspecific secondary antibody signals, negative controls were prepared by staining both control and treated sections with secondary antibodies alone.

### Confocal imaging and analysis

Immunostained sections were visualized using laser scanning confocal microscopy (TCS SP5, Leica Microsystems). To prevent spectral overlap and signal bleed-through, images were acquired in sequential scanning mode. High-magnification images (40X) of sciatic nerve sections were processed using I.A.S. software (Delta Systems, Italy). Fluorescence intensity of IRS1 protein was quantified using ImageJ software (version 1.41, National Institutes of Health, USA). At least two sections per nerve were analyzed, with fluorescence quantification performed by converting pixel intensity into brightness values using the RGB (red, green, and blue) method. This approach allows for the digital detection and analysis of biological samples in confocal microscopy (22).

### Body temperature measurement

Core body temperature (BT) was recorded rectally using a digital thermometer with a precision of 0.1 °C. Measurements were taken under baseline (BL) conditions and after 5 h of exposure to a cold environment (4 °C) across all experimental groups. The same procedure was repeated in the same animals seven days post-injury (D7) for both male and female mice.

### Energy metabolism assessment

Energy expenditure (EE), metabolic rate (MR), oxygen consumption (VO_2_), and respiratory quotient (RQ) were assessed using an indirect calorimetry (IC) system (TSE PhenoMaster/LabMaster System®) with a constant airflow rate of 0.35 L/min. Mice (*N* = 9–11 per group) were acclimated to the metabolic chamber for 6 h before data collection. VO_2_ was recorded every 20 min for individual mice, starting at 7:00 PM and continuing automatically for 48 h to allow comparison between the dark and light phases of the cycle. Room temperature was maintained at a constant 22 °C (±1 °C). The respiratory exchange ratio (RER), calculated as the ratio of CO_2_ produced to O_2_ consumed (RER = VCO_2_/VO_2_), served as an indicator of substrate utilization. EE was determined using the formula: EE = (3.815 + 1.232 × VCO2/VO2) × VO2EE = (3.815 + 1.232 × VCO_2_/VO_2_) × VO_2_EE = (3.815 + 1.232 × VCO2/VO2)  ×  VO2 as provided by the TSE System. EE and RER were analyzed across the entire 48-hour recording period, with specific attention to animals' resting conditions (i.e., values recorded during activity counts between 0 and 3). Locomotor activity was evaluated during the IC session by tracking the number of infrared beam interruptions. Each cage in the IC system was equipped with the InfraMot® device, which utilizes passive infrared sensors to detect and record mouse movement by capturing body-heat images and monitoring spatial displacement over time. All forms of body movement were detected and recorded as activity data.

### Targeted metabolomics by flow injection analysis–tandem mass spectrometry (FIA-MS/MS)

Dried blood spot (DBS) samples were collected by spotting whole blood from each mouse onto a filter paper card. The metabolic profile, including 18 amino acids (AAs), free carnitine (C0), and 30 acylcarnitines (ACCs), was analyzed using Flow Injection Analysis–Tandem Mass Spectrometry (FIA-MS/MS) with the NeoBase Non-Derivatized MSMS Kit (PerkinElmer Life and Analytical Sciences, Turku, Finland) (3, 18, 23–27). For metabolite quantification, isotopically labeled internal standards (ISs) were added to each analyte before extraction. Disks of 3.2 mm (corresponding to 3.0–3.2 μl of whole blood) were punched out from DBS samples and quality controls (QCs) and placed into a polypropylene microtiter plate. An extraction solution containing ISs (100 μl) was then added to each well. The ISs, along with the extraction solution and QCs, were provided in the NeoBase Kit (PerkinElmer Life and Analytical Sciences, Turku, Finland). The microplate was sealed and shaken at 700 rpm for 50 min at 45 °C in a thermo mixer. Subsequently, 75 μl of the supernatant from each well was transferred to a new microplate and loaded into the autosampler for analysis. Both low and high QCs were processed in duplicate under identical conditions as the experimental samples. Metabolic profiling of DBS samples was conducted using a Liquid Chromatography Tandem Quadrupole Mass Spectrometry (LC-MS/MS) system (Alliance HT 2795 HPLC Separation Module coupled to a Quattro Ultima Pt ESI, Waters Corp., Manchester, UK). The mass spectrometer operated in positive electrospray ionization (ESI) mode, employing multiple reaction monitoring (MRM) for data acquisition. MassLynx V4.0 software (Waters Corp.) was used for instrument control, while data processing was automated via NeoLynx (Waters Corp.). Sample injections (30 μl) were introduced directly into the ion source through a narrow PEEK tube, with an injection-to-injection cycle of 1.8 min. The ionization source parameters were optimized to maximize ion yield for each metabolite, with the following settings: capillary voltage at 3.25 kV, source temperature at 120 °C, desolvation temperature at 350 °C, and collision cell gas pressure at 3–3.5 × 10⁻³ mbar (Argon) (18, 23–27).

The list of analyzed metabolites and a description of abbreviations as used in the text are available in Table 1.

**Table 1:** FIA-MS/MS acquisition parameters used for the determination of whole blood amino acids (AAs) and acylcarnitines (ACCs). MS/MS transitions for each analysed AA and ACC and the corresponding internal standard (IS, shown in bold), the optimal cone potential (V), and collision energy (eV) are shown for each analyte. The capillary potential was 3.5 kV.

Abbreviation	Full name	Transition	Cone potential	Collision energy
IS				
Ala	Alanine	90.2 > 44.3	40	6
		94.2 > 48.3		
D4Ala				
Arg	Arginine	175.3 > 70.3	45	17
His	Histidine	156.2 > 110.2		
		180.3 > 75.3		
D5Arg				
Cit	Citrulline	176.2 > 113.2	45	13
D2Cit		178.2 > 115.2		
Gly	Glycine	76.2 > 30.3	40	5
D2Gly		78.2 > 32.3		
Leu/Ile/Pro-OH	Leucine/Isoleucine/Hydroxyproline	132.2 > 86.3	40	8
Asp		134.2 > 88.2		
Glu	Aspartic Acid	148.2 > 84.2		
Asn	Glutamic Acid	133.2 > 74.2		
D3Leu	Asparagine	135.2 > 89.3		
Met	Methionine	150.2 > 104.2	45	9
D3Met		153.2 > 107.2		
Orn	Ornithine	133.3 > 70.3	40	12
Lys/Gln	Lysine/Glutamine	147.2 > 130.2		
D6Orn		139.3 > 76.3		
Phe	Phenylalanine	166.2 > 120.2	45	11
D6Phe		172.2 > 126.2		
Tyr	Tyrosine	182.2 > 136.2	45	12
D6Tyr		188.2 > 142.2		
Val	Valine	118.2 > 72.3	40	8
Ser	Serine	106.1 > 60.3		
Thr	Threonine	120.2 > 74.3		
D8Val		126.2 > 80.3		
C0	Free Carnitine	162.2 > 103.2	60	14
D9C0		171.2 > 103.2		
C2	Acetylcarnitine	204.2 > 85.2	60	14
D3C2		207.2 > 85.2		
C3	Propionylcarnitine	218.2 > 85.2	60	15
D3C3		221.2 > 85.2		
C4 C3DC/C4OH	Butyrylcarnitine	232.3 > 85.2	60	15
D3C4	Malonylcarnitine/3-Hydroxy-butyrylcarnitine	248.3 > 85.2		
		235.3 > 85.2		
C5 C5:1 C4DC/C5OH	Valerylcarnitine	246.2 > 85.2	70	16
D9C5	Tiglylcarnitine	244.2 > 85.2		
	Methylmalonylcarnitine/3-Hydroxy-valerylcarnitine	262.2 > 85.2		
		255.2 > 85.2		
C6 D3C6	Hexanoylcarnitine	260.3 > 85.2	65	16
		263.3 > 85.2		

### Label-Free differential proteomic shotgun analysis

Proteomic analysis was conducted on total proteins extracted from AT collected from four experimental groups (*n* = 3 mice per pool, with three technical replicates per group). The groups consisted of male and female mice at baseline (BL) and seven days post-injury (D7). AT was dissected from the subcutaneous abdominal (inguinal) adipose panniculus. Protein concentration was determined, and 50 μg of total protein from each sample was processed using the filter-aided sample preparation (FASP) protocol, which integrates protein purification and enzymatic digestion, as previously described (28). Samples were transferred onto a Microcon-10 Centrifugal Filter with a 10 kDa molecular weight cut-off (Millipore). After buffer exchange with Urea Buffer (UB: 8M urea, 100 mM Tris-HCl, pH 8.5), proteins were denatured, reduced in 8 mM dithiothreitol (DTT) for 15 min at 56 °C, and alkylated with 0.05M iodoacetamide (IAA) for 20 min at room temperature. Prior to digestion, the buffer was exchanged with 0.05 M ammonium bicarbonate (AmBic). Trypsin digestion was performed at an enzyme-to-protein ratio of 1:50 (w/w) for 16–18 h at 37 °C. The reaction was halted by adding formic acid (FA) to a final concentration of 0.2% (v/v), and peptides were recovered in 0.05 M AmBic, concentrated using a speedvac, and stored at −80 °C until further analysis. After digestion, peptides were resuspended in 0.1% FA, and 0.300 µg of each sample was spiked with 300 fmol of MassPREP Enolase Digestion Standard (Waters Corp.) as an internal reference. Each sample was analyzed in four technical replicates. Tryptic peptides were separated using an ACQUITY M-Class System (Waters Corp.). A 3 µl aliquot of the digested sample was injected onto a Symmetry C18 5 μm, 180 μm × 20 mm precolumn (Waters Corp.), followed by separation using a 90 min reversed-phase gradient (2%–40% acetonitrile over 75 min) at a flow rate of 300 nl/min on an HSS T3 C18 1.8 μm, 75 μm × 150 mm nanoscale LC column (Waters Corp.) maintained at 40 °C. Mobile phase A consisted of 0.1% FA in water, while mobile phase B contained 0.1% FA in acetonitrile (ACN). Peptide analysis was performed using a High-Definition Synapt G2-Si Mass Spectrometer (Waters Corp.), coupled directly to the chromatographic system. Data acquisition followed a data-independent acquisition (DIA) workflow (MSE mode). The instrument was operated with the following settings: electrospray ionization in positive mode (ES+), mass range acquisition of 50–2,000 m/z, capillary voltage of 3.2 kV, source temperature of 80 °C, cone voltage of 40 V, time-of-flight (TOF) resolution of 20,000, precursor ion charge state range of 0.2–4, trap collision energy of 4 eV, transfer collision energy of 2 eV, precursor MS scan time of 0.5 s, and fragment MS/MS scan time of 1.0 s Lock mass correction was performed post-acquisition using the doubly charged monoisotopic ion of [Glu1]-Fibrinopeptide B (Waters Corp.), sampled every 30 s. Continuum LC-MS data from three replicate runs per sample were analyzed for qualitative and quantitative assessment using Progenesis QI for Proteomics v4.1 (Nonlinear Dynamics, Waters Corp.). Protein identification was performed by searching against the Mus musculus database (UniProt.Swiss-Prot release 2021_04), with the sequence of Enolase 1 from *Saccharomyces cerevisiae* (UniProtKB/Swiss-Prot AC: P00924) appended for absolute quantification. Search parameters included:

• Trypsin as the specified digestion enzyme
• Automatic tolerance for precursor and product ions
• Minimum of 3 fragment ions matched per peptide
• Minimum of 7 fragment ions matched per protein
• At least 1 peptide matched per protein
• Allowance of 1 missed cleavage
• Carbamidomethylation of cysteine as a fixed modification
• Oxidation of methionine as a variable modification
• False discovery rate (FDR) set at ≤1% at the protein level

For quantitative analysis, the “Absolute Quantitation Using HiN” option in Progenesis QI software was used, with MassPREP Enolase Digestion Standard as an absolute calibrant. The three most abundant peptides per protein (*N* = 3) were measured for quantification (28). Mass spectrometry proteomics data were deposited in the **ProteomeXchange Consortium** via the **PRIDE** repository with dataset identifier **PXD061152** (DOI: 10.6019/PXD061152).

### Serum steroid profiling by UPLC-MS/MS

Corticosterone (CCONE), 11-deoxycortisol (11-DECOL), dehydroepiandrosterone (DHEA), dehydroepiandrosterone sulfate (DHEAS), 4-androstene-3,17-dione (ADIONE), testosterone (TESTO), 17α-hydroxyprogesterone (17-OHP), and progesterone (PROG), along with their corresponding isotopically labeled internal standards (2H8-CCONE, 2H5-11-DECOL, 2H6-DHEAS, 2H5-ADIONE, 2H5-TESTO, 2H8-17-OHP, 2H9-PROG), were obtained from the CHSTM MSMS Steroids Kit (PerkinElmer®, Turku, Finland). Blood samples (approximately 200 μl per mouse) were collected at BL and seven days post-nerve lesion via decapitation. Samples were kept at room temperature (23 ± 1 °C) to allow coagulation, then centrifuged at 4 °C for 15 min at 1,400 g. Serum was carefully collected, avoiding the layer immediately above the buffy coat, and transferred into polypropylene tubes. Each endogenous steroid (1 mg) was dissolved in ethanol and stored at −20 °C. Stock solutions were diluted in a methanol/water (50:50) solution to obtain a final concentration of 0.03 μM (tuning solution). The internal standard (IS) mix provided in the PerkinElmer® kit was reconstituted in 1.25 ml of acetonitrile (ACN). A daily Precipitation Solution (DPS) containing ISs was prepared by diluting the IS mix 1:100 in ACN with 0.1% FA. Calibrators and quality control (QC) samples, derived from human serum, were obtained from the CHSTM MSMS Steroids Kit (PerkinElmer®, Turku, Finland). The concentration levels (ng/ml) of each steroid analyzed in the LC-MS/MS method are summarized in Table 2.

**Table 2:** MS/MS operating conditions. Multiple reaction monitoring (MRM) functions and settings for detection of steroids are shown. Italics denotes qualifier ion.

MRM Function	Time Window (min)	Analyte	Transitions (*m*/*z*)	Cone Volts	Coll Energy (eV)
1	5.0–8.0	CCONE	346.98 > 121.08	38	24
		*CCONE*	*346.98* *>* *97.09*	*38*	*24*
		^2^H_8_-CCONE	354.98 > 125.08	38	24
2	5.0–8.0	11-DECOL	346.98 > 109.06	40	28
		*11-DECOL*	*346.98* *>* *97.09*	*40*	*28*
		^2^H_5_-11-DECOL	351.98 > 100.09	40	28
	4.25–7.0	DHEAS	271.2 > 197.1	32	18
		*DHEAS*	271.2 > 213.2	32	18
		^2^H_6_-DHEAS	277.1 > 219.2	32	18
	6.0–9.5	DHEA	271.2 > 197.1	32	17
		*DHEA*	271.2 > 213.2	32	17
		^2^H_8_-OHP			
3	5.5–8.5	ADIONE	287.04 > 97.03	36	24
		*ADIONE*	*287.04* *>* *109.06*	*36*	*24*
		^2^H_5_-ADIONE	292.04 > 100.03	36	24
4	6.0–9.0	TESTO	289.04 > 97.09	38	26
		*TESTO*	*289.04* *>* *109.05*	*38*	*26*
		^2^H_5_-TESTO	294.04 > 100.09	38	26
5	7.5–9.5	17-OHP	331.04 > 97.09	40	32
		*17-OHP*	*331.04* *>* *109.06*	*40*	*32*
		^2^H_8_- OHP	339.04 > 100.09	40	32
7	7.5–10.5	PROG	315.204 > 97.09	40	24
		*PROG*	*315.04* *>* *109.05*	*40*	*24*
		^2^H_9_-PROG	324.204 > 100.09	40	24

### Data analysis

All results are presented as mean ± SEM. The sample size for *in vivo* experiments was determined in advance using Power analysis (G*Power 3.1). Statistical analyses were selected based on the nature of the data. Depending on the dataset, comparisons were conducted using either an unpaired t-test, one-way analysis of variance (ANOVA), or two-way repeated-measures ANOVA. For experiments with small sample sizes (*N* < 5 animals) and groups larger than three, non-parametric analysis was performed using the Kruskal–Wallis test. In cases of multiple comparisons, *post-hoc* analysis was conducted using the Tukey-Kramer test, while *t*-tests were applied for single comparisons. Statistical significance was set at *P* < 0.05. Statistical analyses were carried out using Statview 5.0, RStudio, and Python. The effects of sex and CCI on whole blood amino acid (AA) and acylcarnitine (ACC) profiles were assessed using two-way repeated-measures ANOVA, followed by Fisher's *post-hoc* test. Sex and CCI were considered independent factors. When data did not meet the assumption of homoscedasticity, ANOVA was performed after Aligned Rank Transformation (ART) for nonparametric factorial analysis. ART allows for nonparametric factorial analyses using standard ANOVA procedures. Statistical significance was considered at *P* < 0.05. For statistical analysis, the following software was utilized: GraphPad Prism 6.0, Statview 5.0, RStudio, Statistica 6.0 (StatSoft, Tulsa, OK, USA), and MetaboAnalyst.

## Results

### Onset and evolution of neuropathy and pain in aging mice

**Figure 2:**
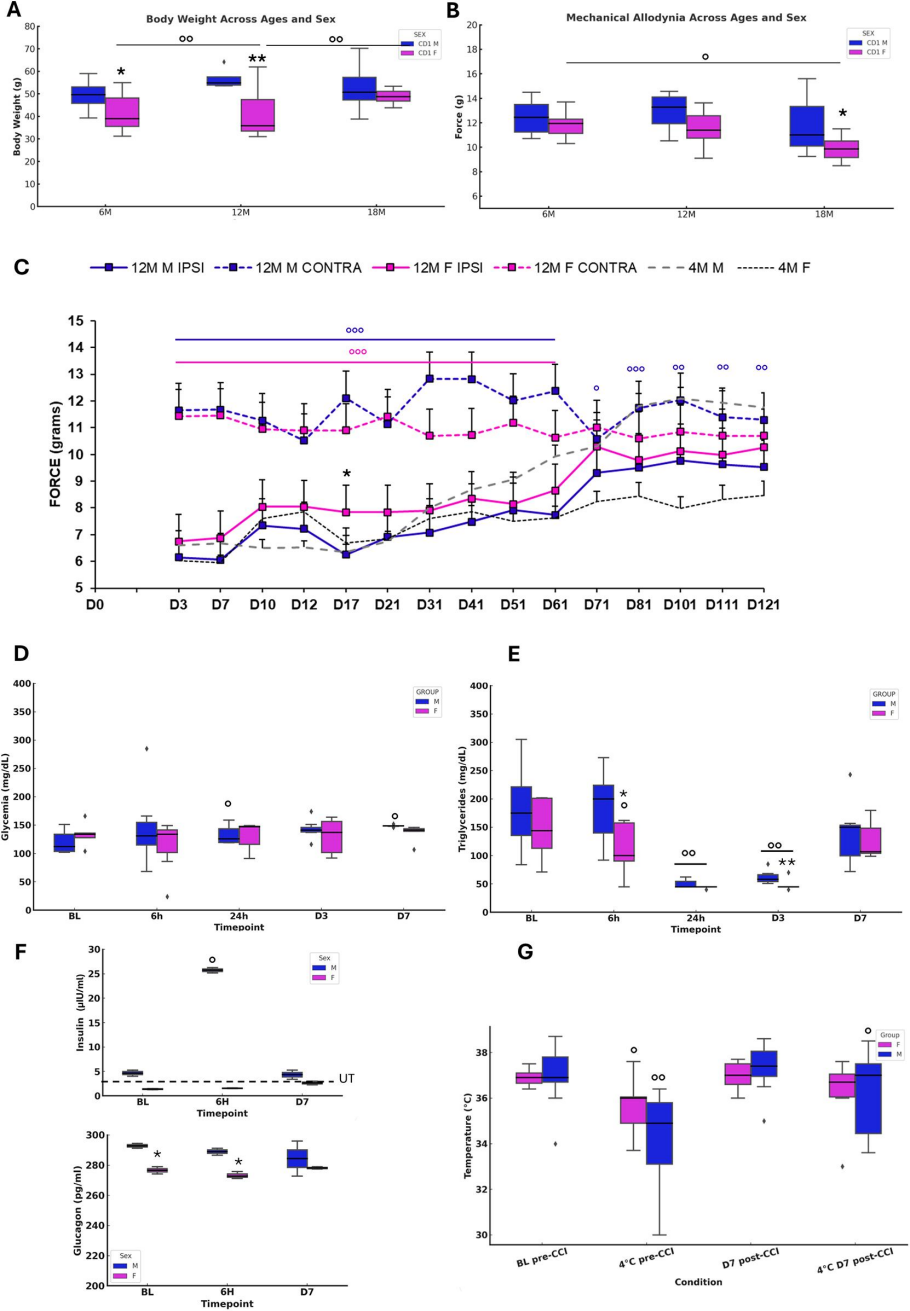
Onset and evolution of neuropathy and metabolic changes in aging mice. **(A)** Body weight in naïve 6M, 12M, 18M animals (*N*=8–10). ANOVA showed a SEX effect (F₁,₂ = 19.647, *p*<0.0001), while AGE (*p* = 0.0823) and SEX × AGE (*p* = 0.0543) were not significant. Tukey tests indicated differences between 6M M vs F (*p* = 0.0272), 12M M vs F (*p* = 0.0008), 6M F vs 12M F (*p* = 0.0015), and 12M vs 18M F (*p* = 0.0394). **(B)** Mechanical threshold (*N* = 8-10) revealed SEX (F₁,₂ = 6.569, *p*=0.0133) and AGE (F₂,₂ = 4.335, *p*=0.0181) effects, but no interaction. Tukey showed differences between 6M vs 18M F (*p* = 0.0371) and 18M M vs F (*p* = 0.0499). **(C)** Mechanical allodynia after CCI in 12M animals (*N* = 11) compared with 4M adults. RM-ANOVA revealed significant SEX (F₃,₄₀ = 371.708, *p* < 0.0001), TIME (F₄₀,₁₄ = 14.227, *p* < 0.0001), and SEX × TIME interaction (F₄₂,₅₆₀ = 9.127, *p* < 0.0001). At D17, unpaired *t*-test confirmed differences between IPSI F vs M (*t*₂₀ = 5.859, *p* < 0.0001). **(D–E)** Glucose and triglycerides after CCI (*N* = 7). Glucose showed increases in males at 24h (*t*₆ = −3.34, *p* = 0.016) and D7 (*t*₆ = −3.73, *p* = 0.0097). Triglycerides showed TIME effects (F₁₂,₄ = 29.138, *p* < 0.0001), with male-female differences at 6h (*t*₁₂ = −2.273, *p* = 0.0422) and D3 (*t*₁₂ = −2.425, *p* = 0.0320), and significant decreases at multiple points in both sexes. **(F)** Insulin/glucagon at BL, 6h, and D7 (*N* = 3). Female insulin remained under threshold, while males showed an increase at 6h (*p* = 0.0495). Glucagon varied significantly (H₅ = 11.556, *p* = 0.0408), with sex differences at BL and 6h. **(G)** Body temperature (12M) showed a cold exposure effect (F₂₀,₃ = 16.875, *p* < 0.0001). Paired *t*-tests revealed decreases after 4 °C in both sexes pre-CCI, and recovery at D7 only in males.

To evaluate the progression of NeP and allodynic response, we used the model of chronic constriction injury (CCI) of the sciatic nerve (21) and assessed the allodynic response via measurement of pain threshold to mechanical stimulation. The investigation of differences in body weight (BW), estrous cycle status, and baseline mechanical thresholds were performed in male and female mice at three distinct ages—6 months (6M, adult and fertile period), 12 months (12M, onset of aging), and 18 months (18M, aging phase). BW increased significantly with age, particularly in males (Figure 2A). Vaginal smear analyses of 6M females confirmed the presence of regular estrous cycles. Cytological analyses in 12M females revealed menopause-like changes in 40% of the animals (Supplementary Table S1). Mechanical thresholds at baseline showed no significant differences between males and females, neither at 6M nor at 12M (Figure 2B). Further analysis, including only the 12M females and the estrous cycle status (cycling vs. non-cycling), indicated no significant effect of the hormonal status on mechanical thresholds (Supplementary Table S1). By contrast, at 18M, a reduction in mechanical threshold was observed in females, and thus the presence of significant allodynia (Figure 2B). The study of NeP progression was performed on 12-month-old male and female mice at the onset of senescence and infertility (peri-menopause/menopause in females). Sensitivity and response to painful stimulation were monitored by the Dynamic Plantar.

Aesthesiometer test continuously for 120 days, including the aging phase (up to 16 months of age) (Figure 2C). Previous studies (10, 11) have highlighted differences in NeP and allodynic response of adult mice subjected to CCI. In our previous study, adult females (4 months old) showed an initial decrease of NeP compared to males but exhibited a chronicization of pain up to 120 days of duration. In contrast, males showed full recovery within 60 days following peripheral nerve injury. Here, in aged subjects, both the onset and progression of allodynia showed a different pattern of expression. The difference between males and females previously observed in adulthood was not evident in aging mice. Both cohorts exhibited heightened sensitivity to mechanical stimulation beginning on day 3 post-injury, followed by a gradual decline in sensitivity. However, especially around day 70 from the CCI, 12M males showed a higher withdrawal response indicative of persistence of NeP, with a trend towards recovery only after day 100. Conversely, aged females showed a complete recovery from NeP from day 70, showing a subsequent complete resolution of chronic pain. The dynamic of functional recovery in older animals suggests sex-specific differences in nocifensive response. Hence, the time-dependent evolution of recovery is different between aged males and females, and in particular, aged male mice exhibited a marked deterioration in recovery time compared to younger males. This prolonged period of recovery in aged males highlights age-related vulnerabilities in the mechanisms of pain resolution. These data reveal that aging significantly affects the trajectory of recovery from NeP, with a protracted persistence of NeP in aged males but with sex-specific differences becoming less marked in older cohorts.

### Sex-dependent metabolic changes following neuropathy

The study explored sex-dependent metabolic and thermoregulatory changes in 12-month-old mice subjected to CCI. The findings report significant differences in metabolic responses to NeP between males and females, underscoring age- and sex-specific adaptations. *in vivo* glycemic levels (Figure 2D) revealed significant sex-specific differences. Aged male mice showed elevated blood glucose levels at 24 h and day 7 (D7) post-CCI compared to baseline (BL), indicating delayed but prolonged glucose dysregulation. In contrast, females maintained stable glycemic levels across all time points, reflecting resilience to alteration of glucose homeostasis following nerve injury. These findings suggest that male mice are more prone to disruption of glucose homeostasis after CCI. Triglyceride levels (Figure 2E) showed different time points across sexes. Males showed significant reductions in triglyceride levels at 24 h and day 3 (D3) post-CCI, with levels returning to baseline by D7. Female mice showed consistent reductions at 6 h, 24 h, and D3 post-CCI. This pattern suggests enhanced lipid clearance in females, in contrast with the delayed and transitory dysregulation observed in males. Insulin levels revealed sex-specific differences (Figure 2F). In female mice, insulin levels were below the manufacturer's minimum detectable threshold, suggesting significant age-related decline in insulin levels (3). Conversely, male mice displayed transient post-CCI hyperinsulinemia at 6 h. Glucagon analysis further revealed sex differences, with significant differences observed at baseline and 6 h post-CCI, reflecting distinct regulatory mechanisms of glucose and energy homeostasis between sexes. Thermoregulatory capacity differed between males and females (Figure 2G). Male mice showed a significant pre-CCI decrease in body temperature during cold exposure, with only partial post-CCI recovery at D7. In contrast, female mice maintained stable body temperature, demonstrating adaptive thermoregulatory mechanisms despite aging and NeP. These findings disclosed a decline of thermoregulatory function in aging males, while females appear to maintain a correct thermoregulation.

### Insulin receptor substrate 1 (IRS1) in sciatic nerve remodeling

IRS1 represents a key downstream mediator of insulin receptor signaling, which has been identified in peripheral nerves and shown to regulate axonal integrity, neuronal survival, and Schwann cell homeostasis (29, 30). Alterations in insulin signaling have been linked to demyelination and Schwann cell de-differentiation, particularly under metabolic stress such as hyperglycemia (31) Based on this evidence, we investigated IRS1 expression in the sciatic nerve to assess whether sex- and age-dependent differences in systemic metabolism are reflected at the level of peripheral nerve insulin signaling. This analysis was aimed at identifying whether IRS1 modulation could contribute to differential vulnerability or resilience to neuropathy between males and females.

**Figure 3:**
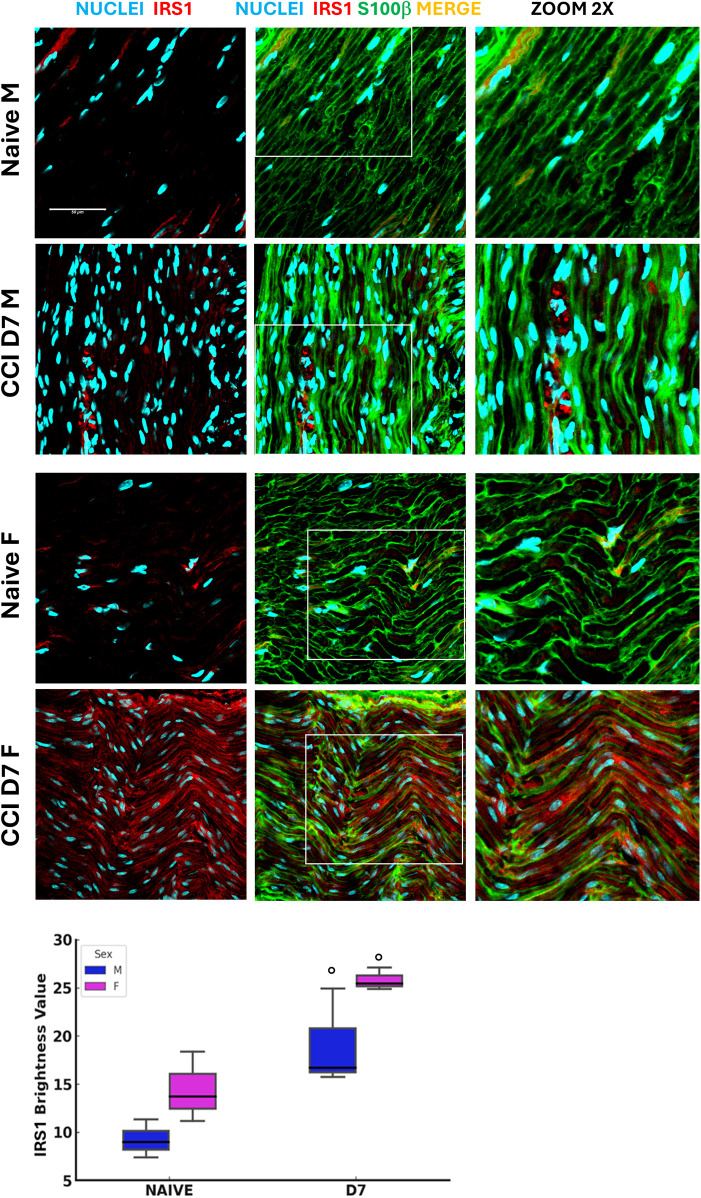
Insulin receptor substrate 1 expression **following neuropathy**. Confocal images (40 × 1.25x) of sciatic nerve sections co-stained for S100*β* (green, Schwann cell marker) and IRS1 (red, Insulin receptor substrate 1) illustrate the distribution of these markers in naïve and CCI (day 7 post-injury) male and female mice (detail in zoom 2x). Scale bar: 50 μm. The graphs show IRS1 expression evaluation in male and female mice at BL and post-CCI (D7) conditions using the RGB analysis, which converts pixels into brightness values. Statistical analysis using the Kruskal–Wallis test (H_3_ = 8.641, *P* = 0.0345) revealed significant differences in IRS1 expression. At BL condition vs. CCI (○) *p* < 0.05 (*N* = 3 per group/time point).

Insulin receptor substrate 1 expression in the sciatic nerve was analyzed under baseline (naïve) and post-CCI conditions (D7) (Figure 3). Confocal imaging revealed distinct patterns of IRS1 localization co-stained with S100 Calcium Binding Protein B (S100β), a Schwann cell (SC) marker. Fluorescence analysis indicated a significant overexpression of IRS1 following CCI in both sexes, suggesting its upregulation as a possible compensatory response to increased energy demand during nerve repair. This response is consistent with whole-body metabolic changes observed in aged mice, emphasizing the role of insulin signaling in peripheral nerve injury and SC function. The sex-dependent differences in metabolism, including glucose dysregulation, lipid changes, and insulin signaling, demonstrate the complex interplay between aging, sex, and neuropathy. While male mice showed prolonged metabolic and thermoregulatory deficits, female mice showed resilient mechanisms to maintain homeostasis. Upregulation of IRS1 in SCs further supports the role of insulin signaling in nerve regeneration, suggesting potential therapeutic targets to address age- and sex-specific metabolic vulnerabilities in the expression of NeP.

### Sex-dependent energy metabolism following neuropathy

**Figure 4:**
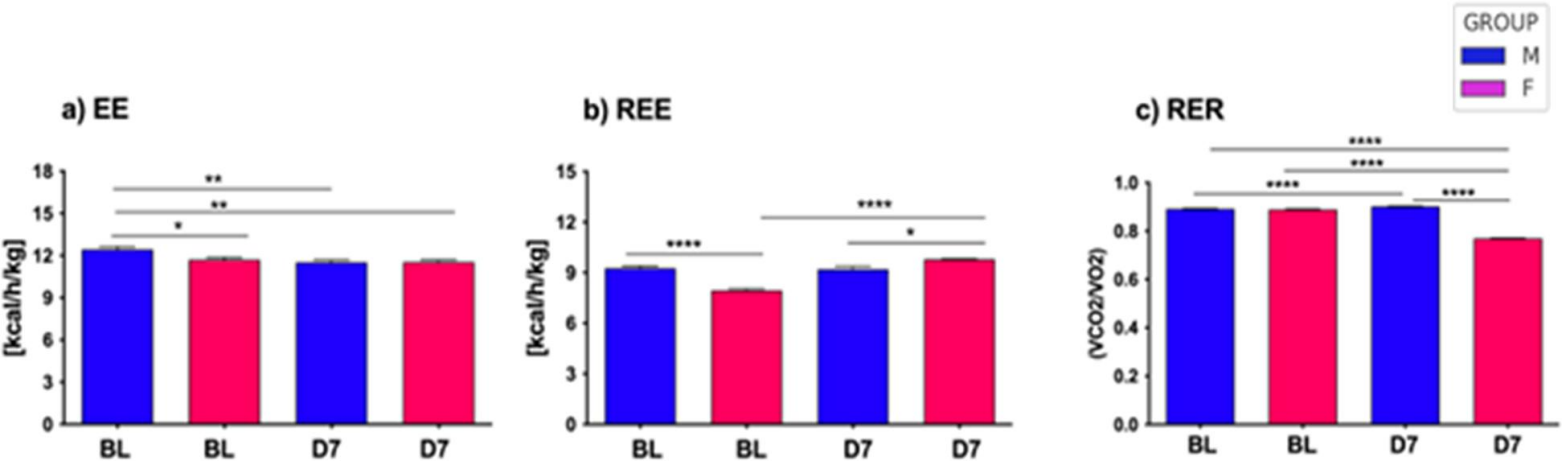
Energy metabolism in aging mice following neuropathy. The left panel **(a)** illustrates the energy expenditure (EE) expressed as kcal/h/kg for both male and female mice at baseline (BL) and day 7 (D7) following chronic constriction injury (CCI). Statistical analysis via two-way ANOVA revealed a significant main effect for CCI/sex factor (F_3,2921_ = 18.74, *p* < 0.0001), and a significant main effect for time factor (F_126_,_2921_ = 18.74, *p* < 0.0001), with further significance between groups observed through Tukey HSD *post-hoc* testing. The middle panel **(b)** shows resting energy expenditure (REE), measured as kcal/h/kg, under similar conditions at BL and D7 after CCI. Two-way ANOVA demonstrated a significant main effect for CCI/sex factor (F_3,276_ = 21.92, *p* < 0.0001), and no effect of time factor (F_171_,_276_ = 1.23, n.s.); significant differences between groups were further confirmed through Tukey HSD *post-hoc* analysis. Lastly, the right panel **(c)** depicts the respiratory exchange ratio (RER), calculated as the VCO_2_/VO_2_ ratio, for both sexes at BL and D7 post-CCI. Statistical evaluation using two-way ANOVA showed a highly significant main effect for CCI/sex factor (F_3_,_2921_ = 18.74, *p* < 0.0001), a significant main effect for time factor (F_126_,_2921_ = 7.63, *p* < 0.0001), with differences between groups validated by Tukey HSD *post-hoc* testing. Statistical significance is indicated as follows: (∗) *p* < 0.005; (∗∗) *p* < 0.01; (∗∗∗) *p* < 0.001; (∗∗∗∗) *p* < 0.0001.

By the indirect calorimetry (IC) analysis, we assessed *in vivo* energy expenditure (EE), resting EE (i.e., in lack of motor activity) (REE), and energy substrate oxidation (respiratory exchange ratio, RER) (Figure 4). Continuous calorimetry recording for 48 h in BL condition disclosed several sex-dependent and CCI-dependent differences in energy metabolism. As compared to male mice, female mice showed lower levels of both EE (panel a) and REE (panel b) at BL, while no differences in RER levels were detected. Peripheral nerve injury at D7 was revealed to induce a decrease of EE in the male mice group, as compared to the pre-CCI (i.e., BL) condition (panel a).

**Figure 5:**
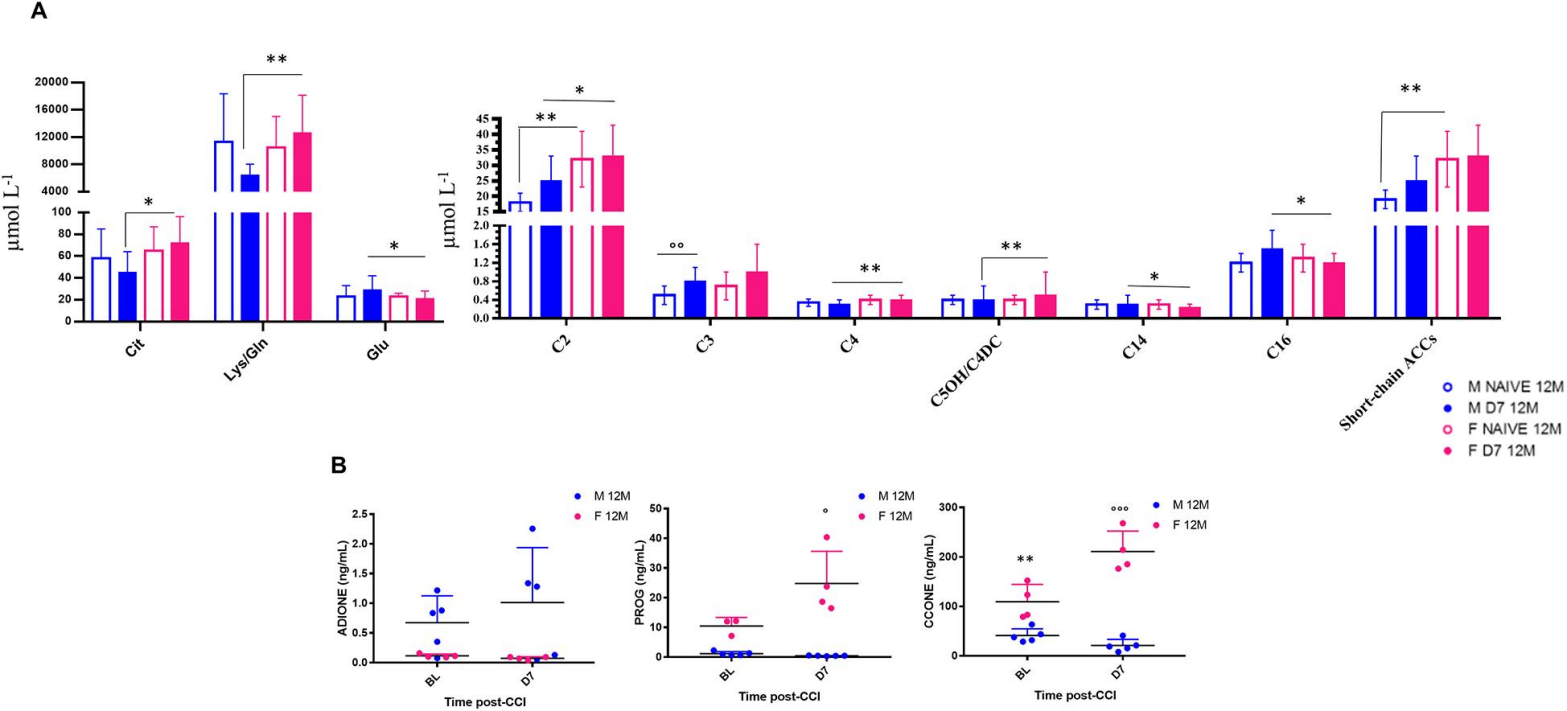
Sex-dependent metabolomic and steroidomic changes in aging mice following neuropathy. Bar graphs represent levels of amino acids (AAs) and acylcarnitines (ACCs) expressed as µmol L^−1^ in 12-month-old male and female mice at baseline (Naïve 12M) and 7 days post-CCI (D7 12M). Table of statistics are reported in S2, and S3. Asterisks (*) indicate significant differences between males and females, while circles (°) denote significant differences between baseline and post-CCI conditions. Statistical significance assessed by two-way ANOVA followed by Fischer's least significance difference (LSD) test is indicated as follows: *p < 0.050, **p < 0.010, ***p < 0.001;°*p* < 0.050,°°*p* < 0.010,°°°*p* < 0.001 (*N* = 7 per group). **(B)** Scatter plots depict the levels of Androstenedione (4-Androstene-7,13-dione) (ADIONE), progesterone (PROG), and corticosterone (CCONE), expressed in ng/ml, in 12-month-old male (blue dots) and female (pink dots) mice at baseline (BL) and 7 days post-CCI (D7). Asterisks (*) indicate significant differences between males and females at baseline, while circles (°) highlight significant differences between baseline and post-CCI conditions (*N* = 4–5 per group) (CCONE: *p* < 0.0001; Tukey's *post-hoc* test *p* = 0.01 for BL males vs. BL females; (Tukey's *post-hoc* test *p* < 0.0001 for BL females vs. D7 females; Tukey's *post-hoc* test *p* < 0.0001 for D7 males vs. D7 females; PROG: Tukey's *post-hoc* test *p* = 0.017 for BL females vs. D7 females; Tukey's *post-hoc* test *p* < 0.0001 for D7 males vs. D7 females.

At difference, peripheral nerve injury (i.e., CCI) did not induce changes in EE in female mice, and levels of EE were not different in female mice between BL and D7 conditions (panel a). On the other hand, female mice showed an increase of REE at D7 as compared to the BL condition (panel b), while such an increase was not detected after CCI in male mice (panel b). Lastly, while no differences were found between male and female mice in BL condition, peripheral nerve damage (i.e., CCI) induced an increase of RER in male mice (BL vs. D7) while a robust decrease of RER in female mice (BL vs. D7). Consequently, male and female mice displayed different RER levels after CCI (D7 male vs. D7 female). These data revealed that peripheral nerve damage decreased whole-body EE only in male mice, while REE was increased after peripheral nerve damage but only in female mice that also displayed lower REE at BL. Such an increase of REE after CCI in female mice appears correlated to the decrease of RER observed in the same mice after peripheral nerve damage. Indeed, peripheral nerve damage appeared to affect the prevalent fuel/energy substrate use, which resulted different between male and female mice. After peripheral nerve injury, the energy substrate oxidation (i.e., RER) switched in female mice from a mixed protein/fatty acids oxidation towards a prevalent fatty acids oxidation, thus revealing a major contribution of fat, rather than carbohydrate, to *in vivo* whole-body energy metabolism. Consequently, nerve injury can alter the glucose-fat metabolism homeostasis and, in females, increase lipid oxidation and EE at rest (REE).

### Metabolomic and steroidomic sex-dependent changes following neuropathy

Targeted metabolomic profiling of 12-month-old mice revealed significant sex- and CCI-dependent changes in amino acid (AA) and acylcarnitine (ACC) levels, both at BL and D7 (Figure 5A, Supplementary Tables S2, S3). At BL, male mice showed lower short-chain ACCs (e.g., C2) levels than females. Following CCI, male mice showed reduced levels of short-chain ACCs (C2, C4, C5OH/C4DC) and longer-chain ACCs (C14, C16) as compared to females. In contrast, male mice exhibited an increase in odd-chain ACCs (C3, C5) at D7, indicating enhanced catabolism of branched-chain amino acids (BCAAs), which may reveal higher energy demand associated with peripheral nerve regeneration. The profile of AA changes also revealed sex- and nerve injury-specific differences. At D7, male mice showed significantly lower levels of citrulline (Cit), lysine/glutamine (Lys/Gln), and glutamate (Glu) compared to females. Notably, Glu, an important excitatory neurotransmitter, was found elevated in male mice post-CCI, suggesting its potential as a biomarker for peripheral nerve injury-induced NeP.

These findings on aging mice (12 months) expand our earlier work on 4-month-old (3), where we used metabolomics on dried blood spot (DBS) to profile a wide range of metabolites, including AAs and ACCs in male and female mice at baseline and 7 days post-CCI (Supplementary Tables S2, S3).

Together, these data underscore sex-specific metabolic adaptations after peripheral nerve injury, with male mice showing exclusive patterns of ACC and AA regulation compared to females, particularly in response to the high metabolic and energy demand induced by peripheral nerve injury. Steroid profiling by UPLC-MS/MS further revealed significant sex differences in glucocorticoids and progesterone (PROG) levels in 12-month-old mice at BL and at D7 post-CCI (Figure 5B, Tables 2, 3). At BL, female mice showed higher levels of corticosterone (CCONE) compared to males, suggesting elevated basal activity of the hypothalamic-pituitary-adrenal (HPA) axis in female mice. After CCI, sex-specific steroid profiles were more evident. Female mice showed a significant increase in CCONE and PROG, indicative of enhanced stress response and potential neuroprotective mechanisms. By contrast, male mice showed significantly lower levels of CCONE and PROG post-injury, suggesting a reduced stress response and impaired metabolic and thermoregulatory adaptation. The increased levels of PROG in females could account for the resilience shown after peripheral nerve injury, as PROG is known for its anti-inflammatory and neuroprotective action. This hormonal effect might contribute to the efficient thermoregulation, as observed in females after CCI. Together, these findings underscore the importance of sex-specific secretion of steroid hormones and the gender differences in the modulation of metabolic responses following damage of peripheral nerve and the development of NeP in aging mice.

**Table 3:** Concentration levels (ng/ml) for calibrators and QC materials of each steroid monitored in the LC-MS/MS method of analysis are summarized.

Analytes	Calibration Levels ng/ml)							QC Levels (ng/ml)		
	*L1*	*L2*	*L3*	*L4*	*L5*	*L6*	*L7*	*QC1*	*QC2*	*QC3*
CCONE	0.29	0.70	1.68	4.03	10.0	24.9	62.0	0.84	4.18	31.0
11-DECOL	0.08	0.20	0.49	1.17	2.91	7.23	18.0	0.24	1.21	9.0
DHEA	0.31	0.73	1.76	4.22	10.5	26.1	65.0	0.88	4.38	32.5
DHEAS	12.9	31.0	74.4	179	444	1,110	2,750	37.2	185	1,380
ADIONE	0.08	0.20	0.49	1.17	2.91	7.23	18.0	0.24	1.21	9.0
TESTO	0.03	0.08	0.20	0.47	1.16	2.89	7.20	0.10	0.49	3.60
17-OHP	0.12	0.29	0.70	1.69	4.20	10.5	26.0	0.35	1.75	13.0
PROG	0.12	0.29	0.69	1.72	4.27	10.6	26.5	0.36	1.78	13.2

### Sex-dependent proteomic changes in adipose tissue following neuropathy

Proteomic profiling of AT revealed distinct sex-specific differences and significant alterations following chronic constriction injury (CCI) in 12-month-old mice. Our experiment was designed to identify the proteins differentially expressed (DEPs) in AT of 12 M-old female and male mice at baseline (BL) or seven days (D7) after sciatic nerve injury (3).

**Figure 6:**
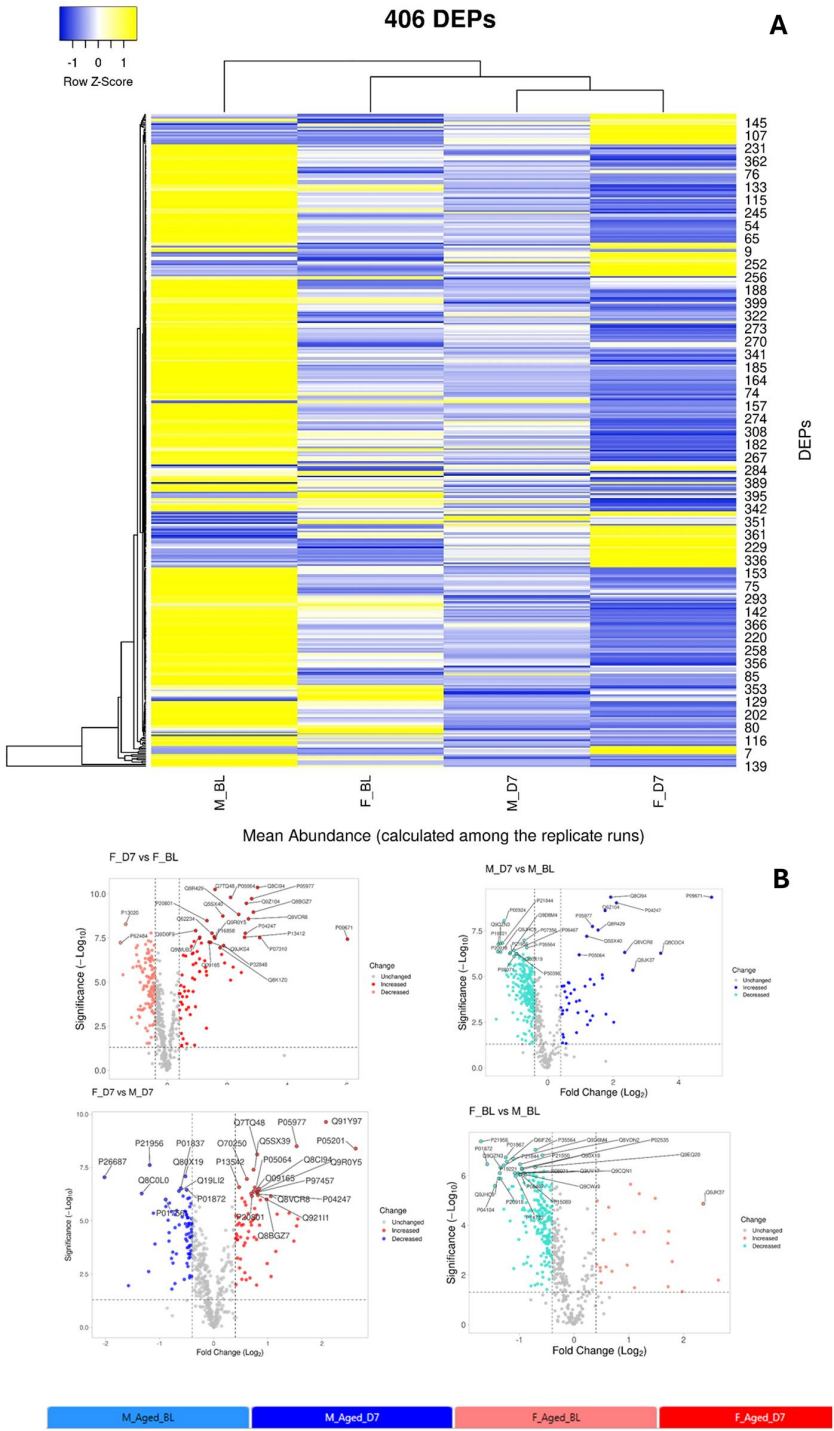
**(A)** Heatmap representation of the 406 differentially expressed proteins (DEPs) across the four experimental groups (M_BL: males in baseline condition; F_BL: females in baseline condition; M_D7: males seven days after CCI; F_D7: females seven days after CCI). The complete list of DEPs is reported in Supplementary Table S4. Rows represent the mean abundance of each protein (measured in three replicate LC-MS runs) in the four groups (*n* = 3 mice per pool, with three technical replicates per group). **(B)** Volcano plots showing the distribution of DEPs for pairwise comparisons:—F_D7 vs. F_BL (top left): females at 7 days post-CCI compared to baseline;—M_D7 vs. M_BL (top right): males at 7 days post-CCI compared to baseline;—F_D7 vs. M_D7 (bottom left): females vs. males at 7 days post-CCI;- F_BL vs. M_BL (bottom right): females vs. males at baseline. On the *x*-axis, the log2 fold-change indicates relative up- or down-regulation of proteins between groups, while the *y*-axis shows statistical significance (–log10 *p*-value). Proteins significantly upregulated are plotted on the right, while significantly downregulated are plotted on the left. Gray dots represent unchanged proteins, while colored dots highlight DEPs: red = increased, blue = decreased, and gray = not significant. The most relevant proteins are annotated with their UniProt IDs. Volcano plots were generated with the VolcaNoseR web application (https://huygens.science.uva.nl/VolcaNoseR/).

This experiment allowed us the quantification of a set of about 500 protein whose expression is differentially modulated in the four groups of mice (females in baseline condition F_BL; females at day 7 after CCI—F_D7; males in baseline condition—M_BL; males at day 7 after CCI M_D7). From this set we selected 406 DEPs with a maximum fold change (MFC) of the protein expression levels set as MFC ≥1.5 in statistically significant observation (ANOVA, *p*-value≤0.05). Details on analysis are reported in the Supplementary Material (Table S4). A heatmap representation (Figure 6A) of the 406DEPs dataset, based on protein recurrence, illustrates the different expression regulation between females and males, which results of particular relevance between BL and D7 (post-injury) in both sexes.

**Figure 7:**
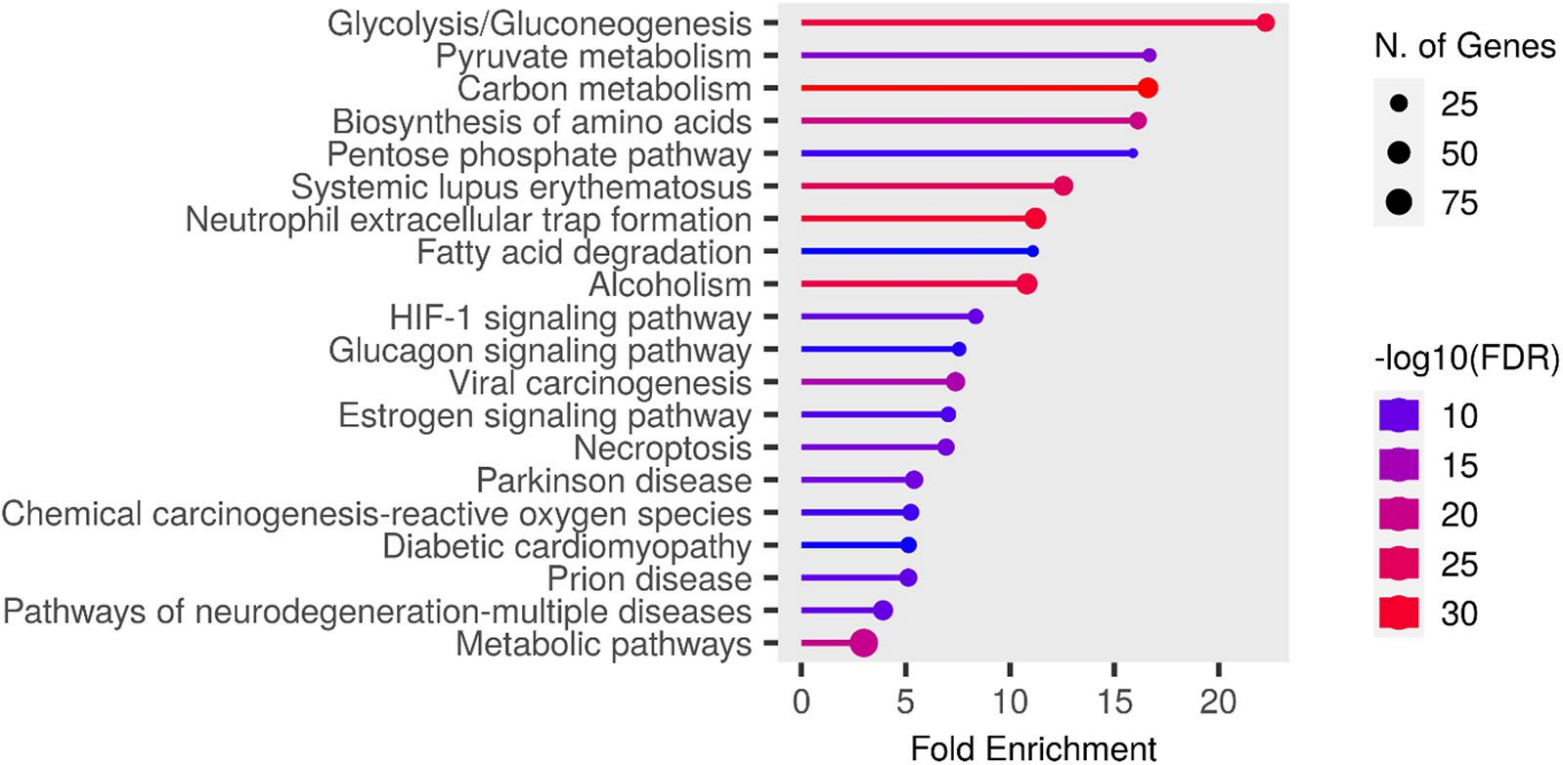
Chart representing enrichment pathway analysis in KEGG pathway database. The chart was obtained using ShinyGO 0.80 (http://bioinformatics.sdstate.edu/go/).

The entire dataset of DEPs underwent pathway enrichment analysis using the ShinyGO tool and the KEGG Pathway database as a reference (32). As illustrated in Figure 7, the majority of proteins whose expression is differentially modulated in both males and females following nerve injury are predominantly associated with pathways involved in energy metabolism.

**Figure 8:**
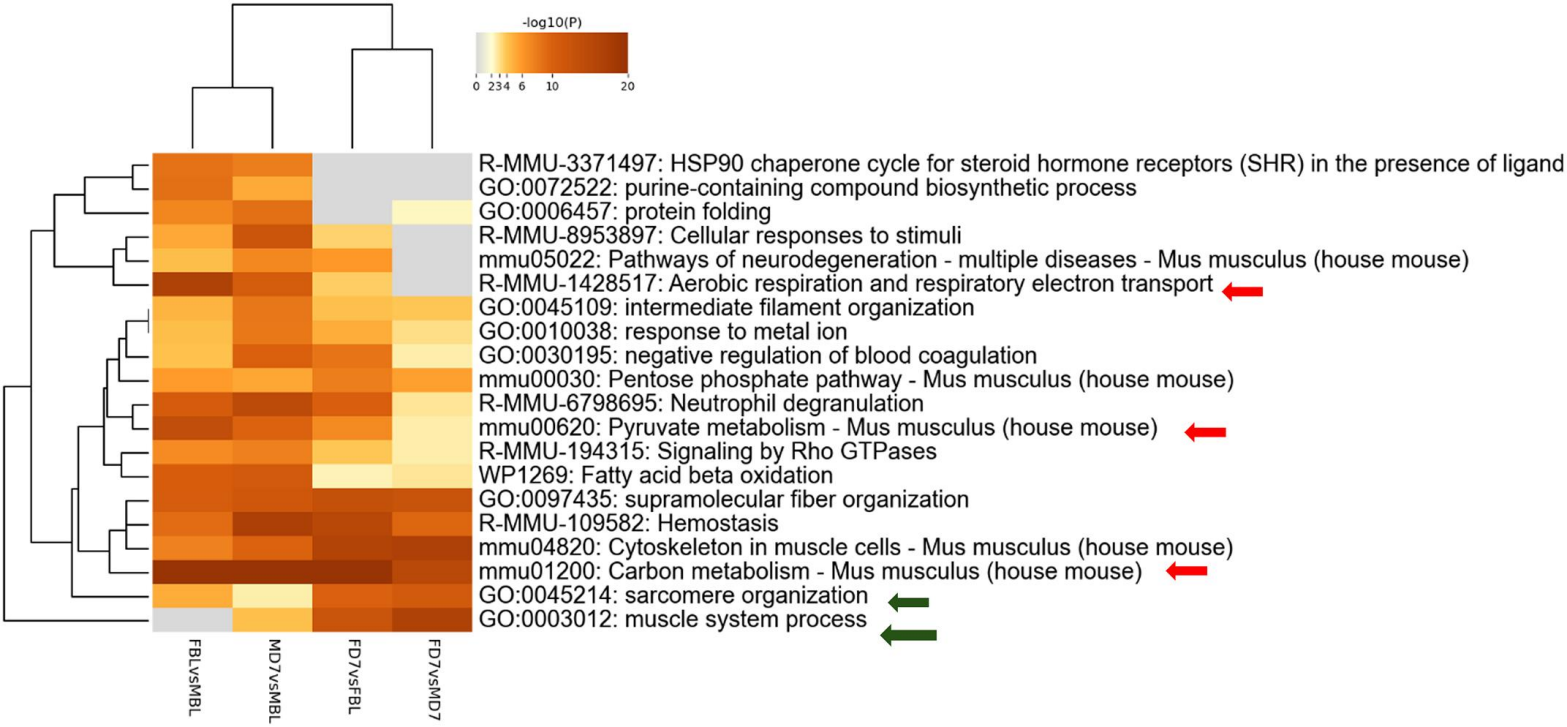
Metascape analysis: enriched terms clusters statistically enriched terms (GO/KEGG terms, reactome canonical pathways, WiKi pathways) are depicted in the heat map. The term with the best *p*-value within each cluster as its representative term are displayed in the dendrogram. The heatmap cells are colored by their *p*-values, white cells indicate the lack of enrichment for that term in the corresponding gene list.

We further analyzed all possible pairwise comparisons among the four experimental groups (Supplementary Table S6). The volcano plots in Figure 6B illustrate statistically significant changes in each comparison, defined by a fold change (FC) ≥ 1.5 and a *p*-value ≤ 0.05 (33). A meta-analysis of these pairing comparisons, using Metascape, a Gene Annotation & Analysis Resource (34), provides an overview of the terms [GO/KEGG terms, Reactome canonical pathways (35), WiKi Pathways] mostly enriched by the proteins modulated across the four conditions: female vs. male at baseline (F_BL vs. M_BL), male and female post-CCI compared to their respective baselines (M_D7 vs. M_BL, F_D7 vs. F_BL), and male vs. female post-CCI (F_D7 vs. M_D7), as shown in Figure 8. The heatmap shown in Figure 6A indicates that the term *carbon metabolism* is enriched across all datasets, though to a lesser extent in comparing males and females post-CCI. A similar trend is observed for aerobic respiration, respiratory electron transport*,* and pyruvate metabolism (red arrow). Additionally, terms associated with muscle regeneration (green arrow) show greater enrichment in the presence of nerve injury.

**Figure 9:**
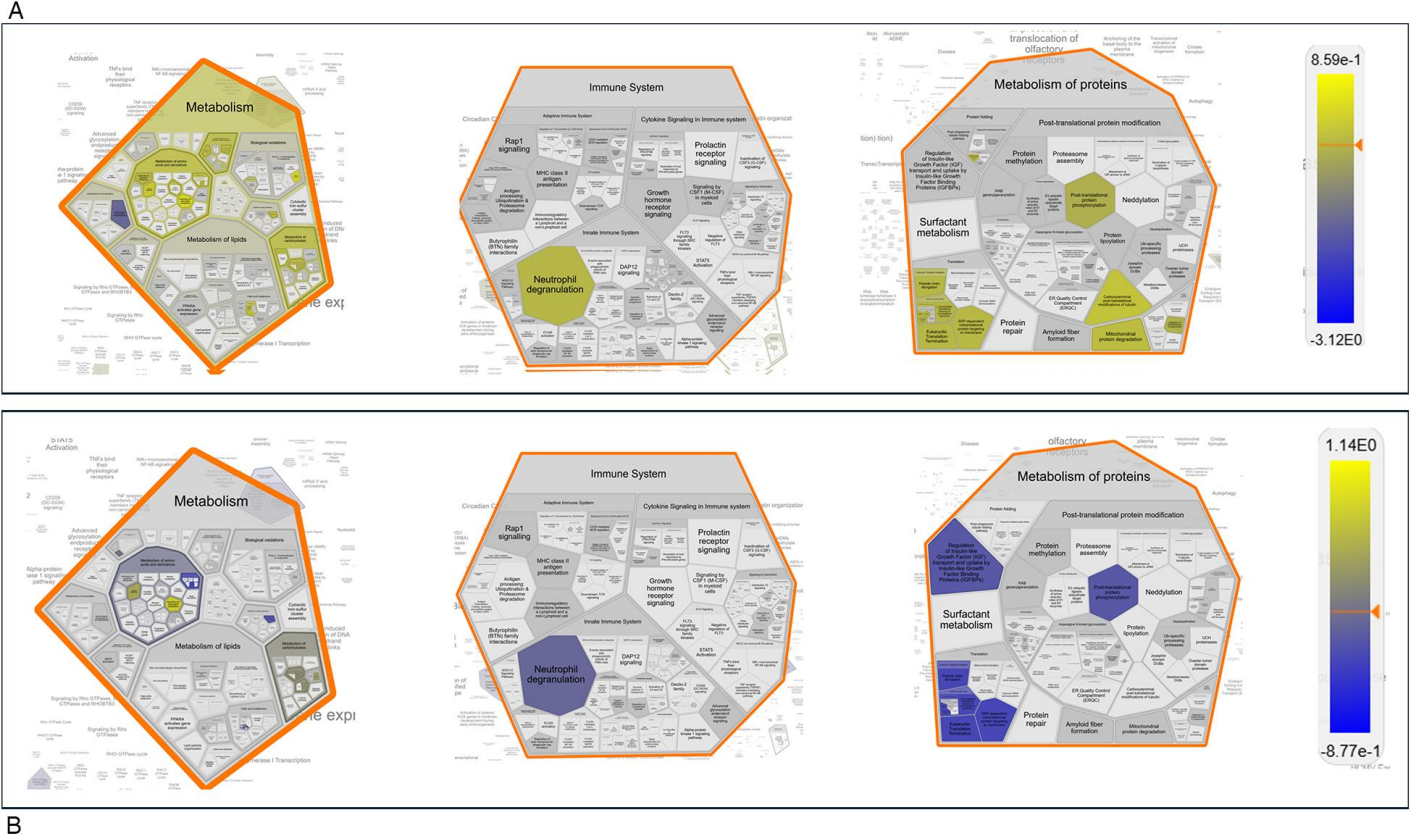
Reactome expression analysis. Voronoi Plot representation of pathways enriched from the differentially expressed proteins (DEPs) with their fold changes in the pairing F_BL vs. M_BL **(A)** and F_D7vs M_D7 **(B)** Protein expression levels in males where always considered as baseline value of expression. The numeric values are used to color objects in pathway diagrams. Yellow indicates positive value of the Log10_FC, hence protein with a higher expression value in female. Blue indicates negative value of the Log10_FC, hence protein with a higher expression value in male The same pathways are more enriched in female at baseline and in males upon nerve injury (post –CCI).

We then focused on sex-related differences in the response to nerve injury by performing an expression analysis using the Reactome database, a platform that allows visualization of expression data mapped onto biological pathway diagrams (35). To explore these differences, we analyzed the lists of differentially expressed proteins (DEPs) and their fold changes from the comparisons F_BL vs. M_BL and F_D7 vs. M_D7 (Table S4 (Datasheet 1)), using males as the baseline reference. The fold change values were used to color-code the elements within the pathway diagrams. As shown in Figure 9, the Voronoi diagram representing all enriched pathways in our datasets highlights those proteins involved in key biological processes, such as Metabolism, Immune System, and Metabolism of Proteins, display opposite regulatory patterns within the same pathways depending on sex. A clear example of this contrasting regulation is observed in the Gluconeogenesis pathway (R-HSA-70263). At baseline, this pathway is primarily enriched with proteins showing higher average expression in males (protein accession numbers: P06745, Q64467, P16858, Q9Z2V4, P21550). However, following nerve injury, the same pathway is instead enriched with proteins exhibiting higher average expression in females (P17751, Q9Z2V4, P09411, P05064, Q91Y97, P05063, P16858, P09041, P21550, O70250). Overall, this analysis indicates that, at baseline, males show greater enrichment of pathways related to lipid metabolism, such as mitochondrial fatty acid oxidation. In contrast, after CCI, females display higher expression of proteins involved in gluconeogenesis (Supplementary Figures S1, S2).

Additionally, in females, we observe increased expression of proteins associated with the Muscle Contraction pathway (R-HSA-397014) following nerve injury—proteins that are not overexpressed in females at baseline (Q5XKE0, P97457, P13412, O09165, P58771, P20801, Q60605, P05977, Q64518, Q8R429, O55143, Q9JI91). This finding may suggest a recovery of muscle strength and activity in the post-injury phase.

### Sex-dependent adipokines and PPARγ changes following neuropathy

Given that AT acts as a key endocrine organ regulating systemic glucose and lipid homeostasis, we next examined the expression of major adipokines (adiponectin and leptin) and their upstream regulator PPARγ. This approach allowed us to link the systemic metabolic alterations observed with potential mechanistic changes within AT that may underlie sex- and age-dependent differences in NeP.

**Figure 10:**
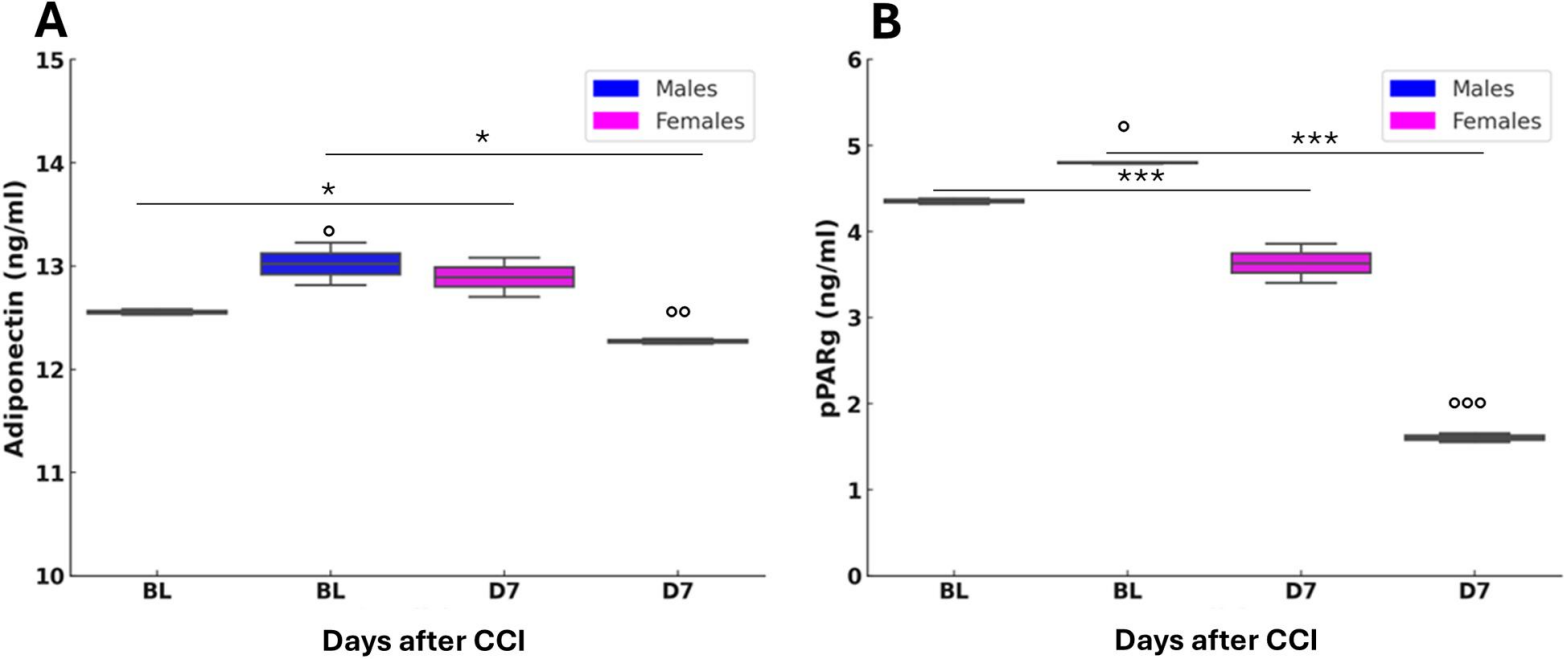
Adiponectin and peroxisome proliferator-activated receptor gamma (pPARγ) variation after nerve lesion in AT. **(A)** Adiponectin measured by enzyme-linked immunosorbent assay (ELISA) in AT at baseline condition (BL) and 7 days after CCI (D7) in male and female mice. Unpaired *t*-test: BL M vs. D7 M *t*_4_ = 3,879, *p* = 0.0179 (*); BL F vs. D7 F *t*_4_ = 5,573, *p* = 0.0051 (*); BL M vs. BL F *t*_4_ = 3,037, *p* = 0.0385 (°); D7 M vs. D7 F *t*_4_ = −6,219, *p* = 0.0034 (°°); *n* = 3 group. **(B)** pPARγ measured by ELISA in AT at baseline condition (BL) and 7 days after CCI (D7) in male and female mice. Unpaired *t*-test: BL M vs. D7 M *t*_4_ = 22,629, *p* < 0.0001 (***); BL F vs. D7 F *t*_4_ = 15.071, *p* = 0.0001 (**); BL M vs. BL F *t*_4_ = −5,452, *p* = 0.0055 (°); D7 M vs. D7 F *t*_4_ = −96,779, *p* < 0.0001 (°°°); *n* = 3 group.

The analysis of circulating levels of leptin showed no significant differences between sexes or between BL and post-CCI D7, indicating that leptin does not play a major role in the sex-specific metabolic response to neuropathy (Supplementary Figure S3). In contrast, adiponectin levels showed significant sex- and CCI-dependent changes. In male mice, adiponectin levels decreased significantly after CCI (Figure 10A), suggesting impaired metabolic regulation and a reduced role of adiponectin signaling in anti-inflammatory activity following nerve injury. In contrast, female mice showed a significant post-CCI increase in adiponectin levels, indicating a potential compensatory mechanism to counteract inflammation and metabolic disruption caused by neuropathy. At baseline, male mice had higher adiponectin levels compared to females, supporting the hypothesis that adiponectin plays a more prominent role in male metabolic homeostasis under physiological conditions. However, following CCI, female mice showed significantly higher adiponectin levels than males, revealing a sex-dependent shift in metabolic and hormonal regulation after peripheral nerve injury. This suggests that females may have an enhanced adaptive response to neuropathy, possibly promoting resilience against metabolic stress. Moreover, different sex-dependent patterns were also observed in pPARγ levels (Figure 10B). In both sexes, pPARγ levels in AT were significantly decreased following CCI, indicating potential dysregulation of pPARγ signaling. The reduction was more pronounced in male mice than in females, suggesting a greater male susceptibility to metabolic imbalance after neuropathy, potentially impairing AT function. Comparisons at BL revealed significantly higher pPARγ levels in male mice than in female mice, reinforcing the idea that pPARγ plays a more central role in male AT homeostasis. After CCI, this sex difference was even more pronounced, suggesting that nerve injury amplifies pre-existing sex differences and makes males more vulnerable to metabolic dysfunction linked to pPARγ loss.

Finally, since the observed sex-specific metabolic signatures could account for differences in macrophage infiltration, we quantified CD11b⁺ cells in the injured sciatic nerve (Supplementary Figure S4) (16, 18, 19). As expected, macrophage density significantly increased at D7 post-CCI in both sexes, with no sex-dependent differences. These findings indicate that local immune activation occurs similarly in males and females at this age and thus are unlikely to underlie the sex-dependent metabolic adaptations observed in AT.

## Discussion

Our study explored sex-specific differences in neuropathy development and chronic pain persistence during aging, with a focus on whole-body and AT metabolism and their contribution to pain processing and recovery following peripheral nerve injury (CCI). While mechanical thresholds and allodynia sensitivity were comparable between sexes at 6 and 12 months, 18-month-old females showed increased sensitivity, suggesting that aging, rather than hormonal fluctuations (in lack of estrous cycle effects) amplifies pain vulnerability in females. Longitudinal monitoring after CCI revealed that females gradually recovered, achieving full resolution by day 70, whereas males displayed sustained hypersensitivity throughout the 121-day observation period. The delayed recovery in males correlated with pronounced metabolic and thermoregulatory deficits. Hyperglycemia was observed only in males at 24 h and 7 days post-CCI, indicating disrupted glucose regulation. Cold exposure before and after CCI revealed that males had lower baseline body temperatures and defective thermoregulatory responses, further deteriorating in post-injury conditions. Conversely, females maintained stable thermoregulation.

The calorimetric analysis provided novel insights into sex-specific energy metabolism following CCI. Females, despite having lower baseline energy expenditure, maintained stable caloric output post-CCI, with a notable increase in resting energy expenditure. This contrasted with the decline observed in males. Additionally, CCI induced a sex-dependent shift in energy substrate use: males slightly increased carbohydrate reliance, while females showed a marked shift toward lipid oxidation, partly explaining their increased energy expenditure. Insulin has a neuroprotective effect on nerve regeneration, and the IR-1-mediated signaling may modulate peripheral nerve injury recovery. The study of diabetic neuropathy in rats has shown that the deficit in IR signaling is associated with exacerbated hyperalgesia (3) or that intrathecal insulin delivery can accelerate axon regeneration and rescue the retrograde loss of collateral axons after peripheral nerve injury (36, 37) Compared to pre-CCI levels, insulin receptor-1 (IR-1) expression increased in male and female mice following experimental neuropathy. These findings suggest that the upregulation of IR-1 is a key mechanism supporting nerve regeneration and recovery from peripheral nerve damage in both sexes, not only in diabetic neuropathy models but also in non-diabetic neuropathy induced by traumatic nerve injury.

Targeted metabolomic profiling revealed robust sex- and CCI-dependent differences in AA and ACC metabolism, highlighting distinct mitochondrial responses to nerve injury. At baseline, female mice showed higher levels of short-chain ACCs, particularly acetylcarnitine (C2), compared to males, suggesting more active fatty acid turnover under physiological conditions. Following CCI, both sexes displayed an overall increase in circulating ACCs, but females maintained significantly elevated C2 levels, along with a marked increase in butyrylcarnitine (C4). In contrast, only males showed an elevation in propionylcarnitine (C3), indicating divergent branched-chain amino acid (BCAA) catabolism between sexes (38, 39). Furthermore, while CCI did not significantly alter long-chain ACCs (e.g., C14, C16) in males, these metabolites were markedly reduced in females. This pattern suggests enhanced mitochondrial fatty acid oxidation in females post-injury, consistent with the observed decrease of RER and shift toward lipid utilization. The reduction in C14 and C16 long-chain ACCs in females may reflect more efficient flux through *β*-oxidation, potentially supported by increased activity of very-long-chain acyl-CoA dehydrogenase, leading to greater conversion into acetyl-CoA and subsequent entry into the tricarboxylic acid cycle. The metabolic shift observed in female mice following CCI, despite stable dietary intake and nutritional status, is reminiscent of a fasting-like state, characterized by increased lipid oxidation, mitochondrial efficiency, and possibly ketogenesis through acetyl-CoA redirection. This adaptation appears protective, as the accumulation of long-chain ACCs like palmitoylcarnitine, is associated with impaired mitochondrial respiration, membrane hyperpolarization, reactive oxygen species (ROS) generation, and mitochondrial damage (40, 41). The reduction of long-chain ACCs in females likely reflects a beneficial mitochondrial response contributing to neuropathy resolution. This metabolic profile may be shaped by sex-specific regulation of *pPAR*γ and adiponectin in AT. Following CCI, males showed increased expression of both markers, while females exhibited a reduction. *pPAR*γ, modulated by nutritional status (42, 43), may be downregulated in response to sustained fatty acid oxidation in females to preserve homeostasis. This mirrors a caloric restriction (CR)-like state, previously associated with improved neuropathy recovery and autophagy activation in wild-type and Ambra1⁺/gt mice (18, 44). Notably, CR-induced reductions in long-chain ACCs in Ambra1 males resembled those observed in aged females after CCI, paralleling their superior functional recovery. The role of adiponectin remains complex: while it exerts anti-inflammatory effects in metabolic disorders (45), it is also associated with increased pain in inflammatory diseases (46–48). Its reduction in females may support higher energy expenditure and thermogenesis (49–52) Additionally, progesterone, elevated in females post-CCI, has neuroprotective and promyelinating effects via metabolites like allopregnanolone (53–56). These hormonal changes may contribute to female resilience, as male mice showed reduced corticosterone and progesterone levels post-injury.

Targeted proteomics of AT revealed 406 differentially expressed proteins (DEPs), with CCI-modulating pathways linked to energy metabolism and muscle function. While pathways related to carbon metabolism, aerobic respiration, respiratory electron transport, and pyruvate metabolism were less represented in females than in males post-CCI, pathways associated with sarcomere organization, muscle system processes, and muscle regeneration were significantly enriched in female mice. Interrogation of the Reactome database disclosed the existence of the gluconeogenesis pathway as particularly enriched by proteins such as beta-enolase (*Eno3*), glyceraldehyde-3-phosphate dehydrogenase (*Gapdh*), glucose-6-phosphate isomerase (*Gpi*) and phosphoenolpyruvate carboxykinase (*Pck1*) that were upregulated only before the CCI. Thus, while males showed early enrichment in gluconeogenesis-related proteins, females displayed post-CCI upregulation of glycolytic and pentose phosphate pathway (PPP) components (e.g., GAPDH, ALDOA, PGK1/2, ENO3, TPI1), which support NADPH production, redox balance, and muscle metabolism (57, 58). This metabolic shift may contribute to the prevention of pentose phosphate pathway (PPP) dysregulation induced by polyol pathway activation under hyperglycemic conditions (31, 59, 60), a transient state observed exclusively in males and known to interfere with SCs function (31, 61). In contrast, the upregulation of enzymes such as GAPDH, PGK1/PGK2, and ENO3 can improve glycolysis efficiency (60), reducing fructose accumulation and preventing excessive ROS production (60, 62). In turn, Aldoa (fructose-bisphosphate aldolase A) can contribute to fructose clearance through glycolysis (63), and GAPDH and PGK enzymes help to regulate NAD+/NADH ratios, potentially offsetting the redox imbalance caused by excessive fructose metabolism (60, 63). Moreover, if TPI1 (triosephosphate isomerase) and PGAM2 (phosphoglycerate mutase 2) are upregulated, fructose-derived intermediates are more efficiently redirected into ATP production rather than accumulating in alternative pathways like the polyol pathway. Moreover, PGK1, PGK2, and GAPDH can regulate glycolysis and impact PPP flux through metabolic rewiring. Upon exposure of cells to oxidative challenge, the mRNA levels of enzymes such as GAPDH can increase and undergo oxidation, leading to the diversion of glucose metabolism towards the PPP to generate NADPH and counteract oxidative stress (58, 62, 63).

Our proteomic data reveal that glycolysis/gluconeogenesis-related proteins are differentially regulated between sexes at baseline and further modulated by CCI in females. The regulation of key enzymes such as PCK1, PFKM, and TALDO1 highlights a coordinated adaptation between glucose and lipid metabolism, potentially reflecting an increased metabolic flexibility of AT after nerve injury. In 12-month-old females, this metabolic program extends beyond glycolytic provision of lipid/steroid precursors to include a coupled glycolysis–pentose phosphate–gluconeogenesis axis and early fatty-acid oxidation, as supported by TALDO1/PCK1 upregulation, respiratory exchange ratio depression, and long-chain acylcarnitine decline. This reconfiguration likely enables a rapid supply of lipid substrates for membrane repair, immune modulation, and steroidogenesis, thereby sustaining post-injury energy demands and favoring resolution of NeP. In contrast, adult females (3) rely mainly on glycolysis-driven lipid handling with limited engagement of fatty-acid oxidation, a profile associated with higher oxidative stress and delayed recovery. Such life-stage– and sex-specific metabolic wiring in AT suggests that carbohydrate–lipid cross-talk, particularly at the PPP–FAO interface and glyceroneogenesis, represents a novel mechanistic axis linking peripheral energy homeostasis to pain chronification, and may constitute an actionable target for tailored interventions in aging and neuropathy.

The ability of female mice to switch between metabolic states likely reflects enhanced metabolic flexibility. Elevated short-chain ACCs, such as C4, combined with reduced RER, indicate preferential lipid utilization and mitochondrial oxidation, simulating exercise or CR-induced adaptations. This profile enables the redirection of glucose intermediates through protective pathways, limiting ROS accumulation and maintaining redox homeostasis. The convergence of molecular, behavioral, and endocrine findings mirrors human epidemiological trends, where midlife males show increased NeP prevalence and metabolic dysfunction (64). In contrast, aged females in our study exhibited improved recovery, driven by efficient lipid oxidation, lower long-chain ACC accumulation, downregulation of the polyol pathway, and sustained thermogenic capacity. These data highlight the central role of sex-dependent metabolic adaptations in determining susceptibility and recovery from NeP. Key regulators such as *pPAR*γ, acylcarnitines, insulin signaling, and steroid hormones emerge as potential therapeutic targets. Notably, pPARγ has already been identified as a mechanistic node in NeP, with several studies demonstrating that its activation alleviates pain and neuroinflammation through modulation of oxidative stress and pro-inflammatory signaling (65–69) as well as *pPAR*γ agonists (70) have shown promise in modulating inflammation in surgical pain models, underscoring the translational relevance of these pathways Our data further extend this concept by linking PPARγ regulation to sex- and age-dependent metabolic reprogramming in AT, suggesting that adipose–neural cross-talk may represent an underappreciated axis in NeP chronification. Altogether, our findings support the development of biomarker-guided, sex- and age-tailored.

## Conclusion: a functional divergence portrayed by metabolic flexibility

Aged females exhibit enhanced metabolic flexibility, defined by increased lipid oxidation, polyol pathway suppression, and improved redox homeostasis, enabling complete NeP recovery. These findings reveal key age- and sex-dependent metabolic adaptations in NeP, with metabolic resilience explaining the enhanced recovery observed in aged females compared to aged males. Peripheral nerve injury imposes substantial metabolic demands, requiring energy-intensive reparative processes. In response, aged females initiate an adaptive metabolic program resembling a fasting state, characterized by preferential lipid utilization, antioxidant defense via the pentose phosphate pathway, neuroprotective steroid biosynthesis, and downregulation of inflammatory and neurotoxic pathways (e.g., the hyper pathway). Conversely, aged males display metabolic inflexibility, accumulation of lipid intermediates, and increased vulnerability to oxidative stress and inflammation. This metabolic divergence between resilience and susceptibility underscores the necessity of age- and sex-specific therapeutic strategies targeting metabolic pathways to enhance neuroprotection and recovery.

## Limitations and future directions

This study did not include the assessment of leptin receptor (LEP-R/OB-R) expression, which we acknowledge as a relevant target for understanding adipokine signaling in the context of age- and sex-dependent neuropathy. Moreover, our analyses were conducted in peri-senescent (12-month-old) mice; future studies will extend these investigations to fully aged (18-month-old) cohorts, where metabolic and hormonal decline is more pronounced. Such work will allow us to verify whether the metabolic configurations identified here—particularly the glycolysis–PPP–lipid remodeling axis in females—persist, amplify, or shift in late aging, and to explore their translational implications. Additional mechanistic endpoints, including receptor profiling, immune–metabolic cross-talk, and longitudinal monitoring of spontaneous pain, should also be incorporated to refine our understanding of sex-specific trajectories in NeP onset and resolution. For istance, IRS1 expression was assessed only in peripheral nerves, whereas its evaluation in dorsal root ganglia and spinal tissues would be essential to fully elucidate the contribution of insulin signaling to NeP mechanisms. Future studies will address this level of analysis to integrate peripheral and central aspects of metabolic regulation.

## Data Availability

The datasets presented in this study can be found in online repositories. The names of the repository/repositories and accession number(s) can be found in the article/Supplementary Material.
