# Supplementary material for: Metabolic resilience governs sex-specific pain recovery during hormonal aging: a multi-omics study of neuropathy in mice
Source: Front Pain Res (Lausanne). 2025 Oct 13;6:1655712. doi: 10.3389/fpain.2025.1655712 (PMC12554762; doi:10.3389/fpain.2025.1655712)
Supplement: Supplementary file 1 [file Table1.docx]

**Table 1. FIA-MS/MS acquisition parameters used for the determination of whole blood amino acids (AAs) and acylcarnitines (ACCs).** MS/MS transitions for each analysed AA and ACC and the corresponding internal standard (IS, shown in bold), the optimal cone potential (V), and collision energy (eV) are shown for each analyte. The capillary potential was 3.5 kV.

| Abbreviation  IS | Full name | Transition | Cone potential | Collision energy |
| --- | --- | --- | --- | --- |
| Ala  D4Ala | Alanine | 90.2>44.3  94.2>48.3 | 40 | 6 |
| Arg  His  D5Arg | Arginine  Histidine | 175.3>70.3  156.2>110.2  180.3>75.3 | 45 | 17 |
| Cit  D2Cit | Citrulline | 176.2>113.2  178.2>115.2 | 45 | 13 |
| Gly  D2Gly | Glycine | 76.2>30.3  78.2>32.3 | 40 | 5 |
| Leu/Ile/Pro-OH  Asp  Glu  Asn  D3Leu | Leucine/Isoleucine/Hydroxyproline  Aspartic Acid  Glutamic Acid  Asparagine | 132.2>86.3  134.2>88.2  148.2>84.2  133.2>74.2  135.2>89.3 | 40 | 8 |
| Met  D3Met | Methionine | 150.2>104.2  153.2>107.2 | 45 | 9 |
| Orn  Lys/Gln  D6Orn | Ornithine  Lysine/Glutamine | 133.3>70.3  147.2>130.2  139.3>76.3 | 40 | 12 |
| Phe  D6Phe | Phenylalanine | 166.2>120.2  172.2>126.2 | 45 | 11 |
| Tyr  D6Tyr | Tyrosine | 182.2>136.2  188.2>142.2 | 45 | 12 |
| Val  Ser  Thr  D8Val | Valine  Serine  Threonine | 118.2>72.3  106.1>60.3  120.2>74.3  126.2>80.3 | 40 | 8 |
| C0 | Free Carnitine | 162.2>103.2 | 60 | 14 |
| D9C0 |  | 171.2>103.2 |  |  |
| C2 | Acetylcarnitine | 204.2>85.2 | 60 | 14 |
| D3C2 |  | 207.2>85.2 |  |  |
| C3 | Propionylcarnitine | 218.2>85.2 | 60 | 15 |
| D3C3 |  | 221.2>85.2 |  |  |
| C4 C3DC/C4OH    D3C4 | Butyrylcarnitine Malonylcarnitine/3-Hydroxy-butyrylcarnitine | 232.3>85.2 248.3>85.2    235.3>85.2 | 60 | 15 |
| C5 C5:1 C4DC/C5OH  D9C5 | Valerylcarnitine Tiglylcarnitine Methylmalonylcarnitine/3-Hydroxy-valerylcarnitine | 246.2>85.2 244.2>85.2 262.2>85.2  255.2>85.2 | 70 | 16 |
| C6 D3C6 | Hexanoylcarnitine | 260.3>85.2 263.3>85.2 | 65 | 16 |
