# Supplementary material for: Metabolic resilience governs sex-specific pain recovery during hormonal aging: a multi-omics study of neuropathy in mice
Source: Front Pain Res (Lausanne). 2025 Oct 13;6:1655712. doi: 10.3389/fpain.2025.1655712 (PMC12554762; doi:10.3389/fpain.2025.1655712)
Supplement: Supplementary file 2 [file Table2.docx]

**Table 2**: Concentration levels (ng/mL) for calibrators and QC materials of each steroid monitored in the LC-MS/MS method of analysis are summarized.

| **Analytes** | **Calibration Levels**  **(ng/mL)** | | | | | | | **QC Levels  (ng/mL)** | | |
| --- | --- | --- | --- | --- | --- | --- | --- | --- | --- | --- |
|  | ***L1*** | ***L2*** | ***L3*** | ***L4*** | ***L5*** | ***L6*** | ***L7*** | ***QC1*** | ***QC2*** | ***QC3*** |
| CCONE | 0.29 | 0.70 | 1.68 | 4.03 | 10.0 | 24.9 | 62.0 | 0.84 | 4.18 | 31.0 |
| 11-DECOL | 0.08 | 0.20 | 0.49 | 1.17 | 2.91 | 7.23 | 18.0 | 0.24 | 1.21 | 9.0 |
| DHEA | 0.31 | 0.73 | 1.76 | 4.22 | 10.5 | 26.1 | 65.0 | 0.88 | 4.38 | 32.5 |
| DHEAS | 12.9 | 31.0 | 74.4 | 179 | 444 | 1110 | 2750 | 37.2 | 185 | 1380 |
| ADIONE | 0.08 | 0.20 | 0.49 | 1.17 | 2.91 | 7.23 | 18.0 | 0.24 | 1.21 | 9.0 |
| TESTO | 0.03 | 0.08 | 0.20 | 0.47 | 1.16 | 2.89 | 7.20 | 0.10 | 0.49 | 3.60 |
| 17-OHP | 0.12 | 0.29 | 0.70 | 1.69 | 4.20 | 10.5 | 26.0 | 0.35 | 1.75 | 13.0 |
| PROG | 0.12 | 0.29 | 0.69 | 1.72 | 4.27 | 10.6 | 26.5 | 0.36 | 1.78 | 13.2 |
