# Supplementary material for: Metabolic resilience governs sex-specific pain recovery during hormonal aging: a multi-omics study of neuropathy in mice
Source: Front Pain Res (Lausanne). 2025 Oct 13;6:1655712. doi: 10.3389/fpain.2025.1655712 (PMC12554762; doi:10.3389/fpain.2025.1655712)
Supplement: Supplementary file 3 [file Table3.docx]

**Table 3**: MS/MS operating conditions. Multiple reaction monitoring (MRM) functions and settings for detection of steroids are shown. Italics denotes qualifier ion.

| **MRM  Function** | **Time Window  (min)** | **Analyte** | **Transitions  (*m*/*z*)** | **Cone  Volts** | **Coll Energy  (eV)** |
| --- | --- | --- | --- | --- | --- |
| 1 | 5.0–8.0 | CCONE | 346.98 > 121.08 | 38 | 24 |
|  |  | *CCONE* | *346.98 > 97.09* | *38* | *24* |
|  |  | ^2^H_8_-CCONE | 354.98 > 125.08 | 38 | 24 |
| 2 | 5.0–8.0 | 11-DECOL | 346.98> 109.06 | 40 | 28 |
|  |  | *11-DECOL* | *346.98 > 97.09* | *40* | *28* |
|  |  | ^2^H_5_-11-DECOL | 351.98 > 100.09 | 40 | 28 |
|  | 4.25-7.0 | DHEAS  *DHEAS*  ^2^H_6_-DHEAS | 271.2>197.1  271.2>213.2  277.1>219.2 | 32  32  32 | 18  18  18 |
|  | 6.0-9.5 | DHEA  *DHEA*  ^2^H_8_-OHP | 271.2>197.1  271.2>213.2 | 32  32 | 17  17 |
| 3 | 5.5–8.5 | ADIONE | 287.04 > 97.03 | 36 | 24 |
|  |  | *ADIONE* | *287.04> 109.06* | *36* | *24* |
|  |  | ^2^H_5_-ADIONE | 292.04 > 100.03 | 36 | 24 |
| 4 | 6.0–9.0 | TESTO | 289.04 > 97.09 | 38 | 26 |
|  |  | *TESTO* | *289.04 > 109.05* | *38* | *26* |
|  |  | ^2^H_5_-TESTO | 294.04> 100.09 | 38 | 26 |
| 5 | 7.5-9.5 | 17-OHP | 331.04 > 97.09 | 40 | 32 |
|  |  | *17-OHP* | *331.04 > 109.06* | *40* | *32* |
|  |  | ^2^H_8_- OHP | 339.04 > 100.09 | 40 | 32 |
| 7 | 7.5-10.5 | PROG | 315.204> 97.09 | 40 | 24 |
|  |  | *PROG* | *315.04 > 109.05* | *40* | *24* |
|  |  | ^2^H_9_-PROG | 324.204> 100.09 | 40 | 24 |
