# Supplementary material for: Metabolic resilience governs sex-specific pain recovery during hormonal aging: a multi-omics study of neuropathy in mice
Source: Front Pain Res (Lausanne). 2025 Oct 13;6:1655712. doi: 10.3389/fpain.2025.1655712 (PMC12554762; doi:10.3389/fpain.2025.1655712)
Supplement: Supplementary file 6 [file Table5.docx]

**Supplementary materials**

**Metabolic resilience rules sex-specific pain recovery during hormonal aging: a multi-omics analysis of neuropathy in mice.**

Sara Marinelli^1‡^*, Claudia Rossi^,3^, Luisa Pieroni^4,5,^ Giacomo Giacovazzo^6,7^, Valentina Vacca^1^, Federica De Angelis^1^, Ilaria Cicalini^2,3^, Valentina Mastrorilli^1^, Chiara Parisi^1^, Zuleyha Nihan Yurtsever^6^, Domenico Ciavardelli^8^, and Roberto Coccurello^6,9‡^*.

^1^National Research Council of Italy (CNR), Institute of Biochemistry and Cell Biology, Monterotondo (RM), Italy.

^2^ Department of Science, "G. d'Annunzio" University of Chieti-Pescara, Chieti, Italy.

^3^Center for Advanced Studies and Technology (CAST), "G. d’Annunzio" University of Chieti-Pescara, Chieti, Italy.

^4^UniCamillus - Saint Camillus International University of Health Sciences,Departmental Faculty of Medicine and Surgery, Rome, Italy.
^5^IRCCS San Camillo Hospital, Venice, Italy

^6^European Center for Brain Research/Institute for Research and Health Care (IRCCS) Santa Lucia Foundation, Rome, Italy.

^7^School of Veterinary Medicine, University of Teramo (UniTE), Italy

^8^School of Medicine and Surgery, “Kore” University of Enna, Enna, Italy.

^9^National Research Council of Italy (CNR), Institute for Complex System (ISC), Rome, Italy.

^‡^Senior author

Lead contact

*Correspondence: Sara Marinelli: [sara.marinelli@cnr.it](mailto:sara.marinelli@cnr.it) telephone: +39 06 90091477**;** Roberto Coccurello: [roberto.coccurello@cnr.it](mailto:roberto.coccurello@cnr.it)

**Table S1. Assessment of the potential impact of the oestrus cycle on neuropathic pain response in 12-month-old mice.**

The fertility status of female mice was evaluated through vaginal smears prior to neuropathy induction to investigate its influence on mechanical threshold sensitivity (measured using an aesthesiometer, with values expressed in grams of force) and body weight (BW, expressed in grams). No significant differences were observed between females in the oestrus cycle and those not cycling, indicating that the fertility status did not affect these parameters.

| STRAIN & SEX | AGE | CYCLE | BW | AE |
| --- | --- | --- | --- | --- |
| CD1 F | 12M | CYCLE | 32,9 | 13,63 |
| CD1 F | 12M | CYCLE | 35,6 | 11,3 |
| CD1 F | 12M | CYCLE | 35,3 | 11,9 |
| CD1 F | 12M | CYCLE | 31 | 9,1 |
| CD1 F | 12M | CYCLE | 32,6 | 11,5 |
| CD1 F | 12M | NO CYCLE | 43,2 | 10,7 |
| CD1 F | 12M | NO CYCLE | 36,2 | 10,86 |
| CD1 F | 12M | NO CYCLE | 51,9 | 12,8 |
| CD1 F | 12M | NO CYCLE | 48,9 | 10,2 |
| CD1 F | 12M | CYCLE | 61,9 | 12,86 |

**Table S2.** **Whole blood** **AAs, C0, and ACCs of male and female mice in BL and D7 condition at 12 months of age (12M).** Data are expressed in μmol L^-1^ extracted sample. Differences between the study groups were assessed by 2-factor ANOVA. p-values lower than 0.050 are shown in bold.

|  | **BL Female, mean (SD)^a^,**  **μmol L^-1^** | | **D7 Female, mean (SD)^a^,**  **μmol L^-1^** | | **BL Male, mean (SD)^a^,**  **μmol L^-1^** | | **D7 Male, mean (SD)^a^,**  **μmol L^-1^** | | **Two way-ANOVA test, p^b^** | | | |
| --- | --- | --- | --- | --- | --- | --- | --- | --- | --- | --- | --- | --- |
| **Metabolite** | | |  |  |  | |  | | **Gender** | | **Time (pre/post CCI)** | **Gender*Time (pre/post CCI)** |
| **C0** | | | 32 (7) | 33 (7) | 28 (8) | | 30 (7) | | 0,20 | | 0,46 | 0,91 |
| **C2** | | | 32 (9) | 33 (10) | 18 (3) | | 25 (8) | | **0,001** | | 0,13 | 0,31 |
| **C3** | | | 0.7 (0.3) | 1.0 (0.6) | 0.5 (0.2) | | 0.8 (0.3) | | 0,28 | | **0,002** | 0,49 |
| **C4** | | | 0.4 (0.1) | 0.4 (0.1) | 0.34 (0.08) | | 0.3 (0.1) | | **0,005** | | 0,93 | 0,28 |
| **C5** | | | 0.21 (0.08) | 0.3 (0.1) | 0.15 (0.07) | | 0.24 (0.08) | | 0,28 | | **0,003** | 0,17 |
| **C4OH/C3DC** | | | 0.20 (0.08) | 0.3 (0.2) | 0.20 (0.09) | | 0.2 (0.1) | | 0,34 | | 0,23 | 0,38 |
| **C6** | | | 0.09 (0.04) | 0.08 (0.03) | 0.08 (0.03) | | 0.08 (0.04) | | 0,72 | | 0,96 | 0,52 |
| **C5OH/C4DC** | | | 0.4 (0.1) | 0.5 (0.5) | 0.4 (0.1) | | 0.4 (0.3) | | **0,002** | | 0,79 | 0,10 |
| **C5DC/C6OH** | | | 0.3 (0.3) | 0.3 (0.1) | 0.17 (0.09) | | 0.3 (0.2) | | 0,55 | | 0,42 | 0,16 |
| **C8** | | | 0.09 (0.02) | 0.08 (0.04) | 0.07 (0.03) | | 0.09 (0.04) | | 0,9 | | 0,81 | 0,17 |
| **C6DC** | | | 0.013 (0.07) | 0.1 (0.1) | 0.2 (0.1) | | 0.2 (0.1) | | 0,17 | | 0,82 | 0,64 |
| **C10** | | | 0.06 (0.03) | 0.06 (0.02) | 0.05 (0.03) | | 0.08 (0.03) | | 0,58 | | 0,18 | 0,24 |
| **C12** | | | 0.08 (0.04) | 0.08 (0.04) | 0.08 (0.03) | | 0.10 (0.05) | | 0,38 | | 0,49 | 0,54 |
| **C14:1** | | | 0.10 (0.05) | 0.09 (0.03) | 0.10 (0.04) | | 0.10 (0.04) | | 0,53 | | 0,86 | 0,97 |
| **C14** | | | 0.3 (0.1) | 0.23 (0.08) | 0.3 (0.1) | | 0.3 (0.2) | | 0,10 | | 0,98 | 0,33 |
| **C14OH** | | | 0.04 (0.02) | 0.05 (0.03) | 0.06 (0.03) | | 0.06 (0.03) | | 0,17 | | 0,95 | 0,95 |
| **C16:1** | | | 0.19 (0.07) | 0.16 (0.05) | 0.21 (0.08) | | 0.20 (0.08) | | 0,086 | | 0,49 | 0,67 |
| **C16** | | | 1.3 (0.3) | 1.2 (0.2) | 1.2 (0.2) | | 1.5 (0.4) | | 0,41 | | 0,48 | 0,052 |
| **C16OH** | | | 0.11 (0.06) | 0.11 (0.06) | 0.14 (0.07) | | 0.16 (0.07) | | 0,064 | | 0,72 | 0,68 |
| **C18:2** | | | 0.21 (0.06) | 0.21 (0.04) | 0.18 (0.05) | | 0.24 (0.09) | | >0.99 | | 0,19 | 0,24 |
| **C18:1** | | | 0.6 (0.1) | 0.6 (0.1) | 0.5 (0.1) | | 0.7 (0.2) | | 0,98 | | 0,28 | 0,2 |
| **C18** | | | 0.4 (0.1) | 0.38 (0.09) | 0.35 (0.08) | | 0.4 (0.1) | | 0,92 | | 0,59 | 0,29 |
| **C18OH** | | | 0.07 (0.04) | 0.07 (0.03) | 0.10 (0.03) | | 0.10 (0.02) | | **0,016** | | 0,49 | 0,79 |

^a^ Standard deviation; ^b^ 95% confidence level; ^c^ 4 months of age; ^d^ 12 months of age.

*(continued)*

|  | **BL Female, mean (SD)^a^,**  **μmol L^-1^** | | **D7 Female, mean (SD)^a^,**  **μmol L^-1^** | | **BL Male, mean (SD)^a^,**  **μmol L^-1^** | | **D7 Male, mean (SD)^a^,**  **μmol L^-1^** | | **Two way-ANOVA test, p^b^** | | | |
| --- | --- | --- | --- | --- | --- | --- | --- | --- | --- | --- | --- | --- |
| **Metabolite** | | |  |  |  | |  | | **Gender** | | **Time (pre/post CCI)** | **Gender*Time (pre/post CCI)** |
| **Short-chain ACCs** | | | 32 (9) | 33 (10) | 19 (3) | | 25 (8) | | **0,001** | | 0,13 | 0,32 |
| **Odd-chain ACCs** | | | 0.9 (0.3) | 1.2 (0.6) | 0.7 (0.2) | | 1.1 (0.4) | | 0,22 | | **<0.001** | 0,33 |
| **3-Hydroxy/Di-Carboxy ACCs** | | | 1.3 (0.4) | 1.4 (0.3) | 1.2 (0.3) | | 1.3 (0.3) | | 0,49 | | 0,38 | 0,9 |
| **Unsaturated chain ACCs** | | | 1.1 (0.3) | 1.1 (0.2) | 1.0 (0.3) | | 1.2 (0.4) | | 0,61 | | 0,55 | 0,33 |
| **Saturated chain ACCs** | | | 2.2 (0.6) | 2.0 (0.3) | 2.0 (0.5) | | 2.5 (0.7) | | 0,38 | | 0,55 | 0,10 |
| **Pro** | | | 48 (17) | 61 (26) | 54 (24) | | 62 (17) | | 0,60 | | 0,18 | 0,74 |
| **Val** | | | 67 (22) | 74 (21) | 65 (18) | | 73 (15) | | 0,75 | | 0,23 | 0,94 |
| **Leu/Ile/Pro-OH** | | | 166 (26) | 177 (51) | 151 (34) | | 198 (39) | | 0,86 | | **0,009** | 0,086 |
| **Orn** | | | 131 (78) | 186 (108) | 198 (129) | | 134 (60) | | 0,78 | | 0,89 | 0,092 |
| **Met** | | | 31 (10) | 36 (21) | 26 (10) | | 30 (13) | | 0,26 | | 0,32 | 0,91 |
| **Phe** | | | 38 (5) | 42 (8) | 34 (6) | | 42 (5) | | 0,37 | | **0,008** | 0,38 |
| **Arg** | | | 65 (19) | 63 (20) | 51 (13) | | 58 (20) | | 0,074 | | 0,73 | 0,46 |
| **Cit** | | | 65 (22) | 72 (24) | 58 (27) | | 45 (19) | | **0,022** | | 0,73 | 0,20 |
| **Tyr** | | | 74 (14) | 78 (16) | 68 (13) | | 88 (23) | | 0,77 | | 0,072 | 0,21 |
| **Gly** | | | 723 (419) | 917 (460) | 1203 (554) | | 857 (468) | | 0,097 | | 0,674 | 0,144 |
| **Ala** | | | 124 (90) | 122 (97) | 146 (94) | | 122 (76) | | 0,65 | | 0,70 | 0,75 |
| **Ser** | | | 4 (2) | 4 (3) | 4 (3) | | 5 (3) | | 0,50 | | 0,44 | 0,96 |
| **Thr** | | | 18 (9) | 17 (7) | 15 (6) | | 19 (9) | | >0.99 | | 0,54 | 0,29 |
| **Asn** | | | 3 (1) | 3.4 (0.8) | 3 (2) | | 4 (2) | | 0,23 | | 0,084 | 0,54 |
| **Asp** | | | 6 (2) | 6 (2) | 6 (1) | | 7 (2) | | 0,90 | | 0,090 | 0,15 |
| **Lys/Gln** | | | 10444 (4606) | 12599 (5540) | 11297 (7061) | | 6417 (1617) | | 0,073 | | 0,46 | 0,068 |
| **Glu** | | | 23 (3) | 21 (7) | 23 (10) | | 29 (13) | | 0,074 | | 0,52 | 0,26 |
| **His** | | | 302 (202) | 311 (142) | 224 (222) | | 288 (159) | | 0,47 | | 0,43 | 0,56 |

^a^ Standard deviation; ^b^ 95% confidence level; ^c^ 4 months of age; ^d^ 12 months of age.

**Table S3.** **Multiple post-hoc comparisons between whole blood AAs, C0, and ACCs of male and female mice in BL and D7 condition at 12 months of age (12M).** The data are p values obtained by Fischer test. p-values lower than 0.050 are shown in bold.

|  | **Fisher post hoc test, p^a^** | | | |
| --- | --- | --- | --- | --- |
|  |  |  |  |  |
| **Metabolite** | **BL Male *vs* D7 Male** | **BL Male *vs* BL Female** | **D7 Male *vs* D7 Female** | **BL Female *vs* D7 Female** |
|  |  |  |  |  |
| **C0** | 0,54 | 0,34 | 0,38 | 0,65 |
| **C2** | 0,082 | **0,004** | **0,048** | 0,71 |
| **C3** | **0,008** | 0,30 | 0,61 | 0,061 |
| **C4** | 0,41 | 0,23 | **0,004** | 0,48 |
| **C5** | **0,003** | 0,17 | 0,89 | 0,18 |
| **C4OH/C3DC** | 0,81 | 0,87 | 0,14 | 0,15 |
| **C6** | 0,68 | 0,53 | 0,91 | 0,63 |
| **C5OH/C4DC** | 0,17 | 0,22 | **0,002** | 0,31 |
| **C5DC/C6OH** | 0,13 | 0,11 | 0,42 | 0,66 |
| **C8** | 0,25 | 0,44 | 0,35 | 0,41 |
| **C6DC** | 0,62 | 0,48 | 0,21 | 0,86 |
| **C10** | 0,080 | 0,43 | 0,13 | 0,89 |
| **C12** | 0,36 | 0,86 | 0,28 | 0,96 |
| **C14:1** | 0,88 | 0,63 | 0,68 | 0,92 |
| **C14** | 0,50 | 0,84 | **0,038** | 0,48 |
| **C14OH** | >0.99 | 0,31 | 0,35 | 0,93 |
| **C16:1** | 0,85 | 0,41 | 0,10 | 0,44 |
| **C16** | 0,063 | 0,24 | **0,027** | 0,35 |
| **C16OH** | 0,59 | 0,27 | 0,12 | 0,97 |
| **C18:2** | 0,083 | 0,40 | 0,40 | 0,92 |
| **C18:1** | 0,099 | 0,22 | 0,23 | 0,88 |
| **C18** | 0,26 | 0,40 | 0,48 | 0,70 |
| **C18OH** | 0,76 | 0,11 | 0,056 | 0,49 |
| **Short-chain ACCs** | 0,083 | **0,004** | 0,045 | 0,70 |
| **Odd-chain ACCs** | **0,002** | 0,21 | 0,62 | **0,039** |
| **3-Hydroxy/Di-Carboxy ACCs** | 0,47 | 0,55 | 0,71 | 0,59 |
| **Unsaturated chain ACCs** | 0,27 | 0,55 | 0,20 | 0,79 |
| **Saturated chain ACCs** | 0,12 | 0,38 | **0,043** | 0,44 |

^a^ 95% confidence level.

*(continued)*

|  | **Fisher post hoc test, p^a^** | | | |
| --- | --- | --- | --- | --- |
|  |  |  |  |  |
| **Metabolite** | **BL Male *vs* D7 Male** | **BL Male *vs* BL Female** | **D7 Male *vs* D7 Female** | **BL Female *vs* D7 Female** |
|  |  |  |  |  |
| **Pro** | 0,46 | 0,53 | 0,91 | 0,24 |
| **Val** | 0,37 | 0,78 | 0,87 | 0,42 |
| **Leu/Ile/Pro-OH** | **0,004** | 0,46 | 0,32 | 0,44 |
| **Orn** | 0,19 | 0,11 | 0,21 | 0,26 |
| **Met** | 0,52 | 0,46 | 0,38 | 0,43 |
| **Phe** | **0,013** | 0,20 | 0,98 | 0,16 |
| **Arg** | 0,44 | 0,062 | 0,50 | 0,78 |
| **Cit** | 0,25 | 0,48 | **0,011** | 0,49 |
| **Tyr** | **0,036** | 0,31 | 0,16 | 0,67 |
| **Gly** | 0,18 | **0,011** | 0,73 | 0,45 |
| **Ala** | 0,61 | 0,53 | 0,99 | 0,96 |
| **Ser** | 0,56 | 0,65 | 0,62 | 0,60 |
| **Thr** | 0,24 | 0,43 | 0,43 | 0,75 |
| **Asn** | 0,10 | 0,60 | 0,24 | 0,41 |
| **Asp** | **0,032** | 0,57 | 0,46 | 0,84 |
| **Lys/Gln** | 0,072 | 0,67 | **0,006** | 0,41 |
| **Glu** | 0,21 | 0,80 | **0,026** | 0,72 |
| **His** | 0,33 | 0,44 | 0,81 | 0,88 |

^a^ 95% confidence level.

**Table S4. DIFFERENTIAL PROTEOMIC ANALYSIS DETAILS.** A total of 406 DEPs were selected from four groups of mice (females in baseline condition – F_BL; females at day 7 after CCI – F_D7; males in baseline condition – M_BL; males at day 7 after CCI – M_D7), with a maximum fold change (MFC) in protein expression levels set at MFC ≥ 1.5 and statistically significant observations (ANOVA p-value ≤ 0.05).
Details of the analysis are provided in the attached Excel file named “Table 4 Differential Proteins”, due to the large size of the dataset, the presence of multiple Excel sheets, and the extensive list of proteins**.**

**Figure S1**. Most Relevant Pathway enriched by the DEPs in females baseline condition vs males baseline condition (FBLvsMBL) sorted by p-value


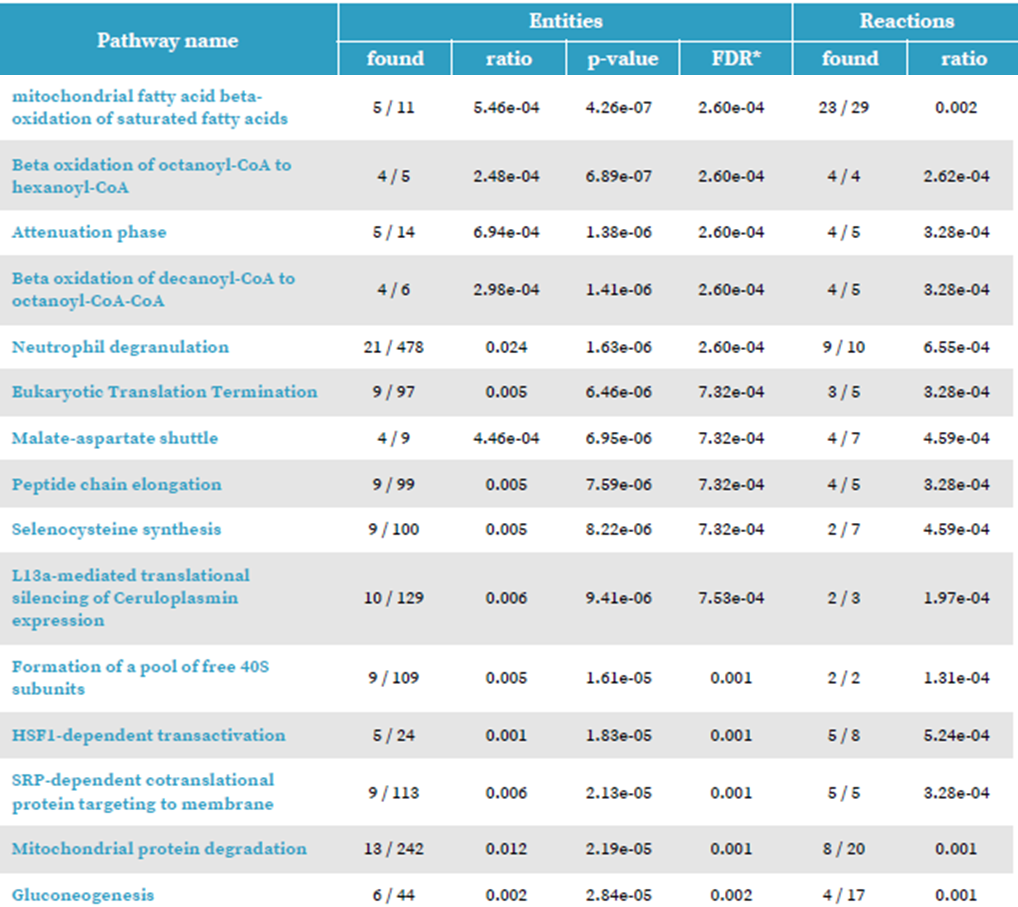


**Figure S2.** Most Relevant Pathway enriched by the DEPs in females 7 days after CCI vs males 7 days after CCI (FD7vsMD7) sorted by p-value


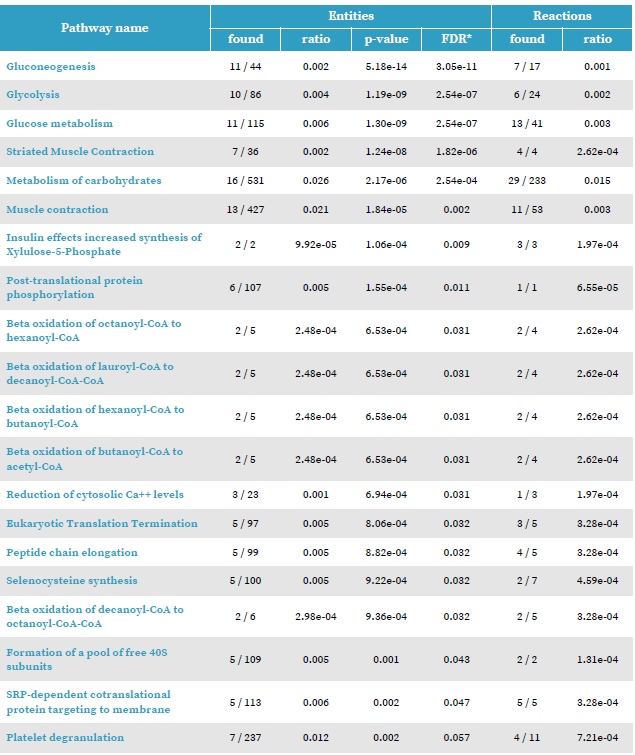


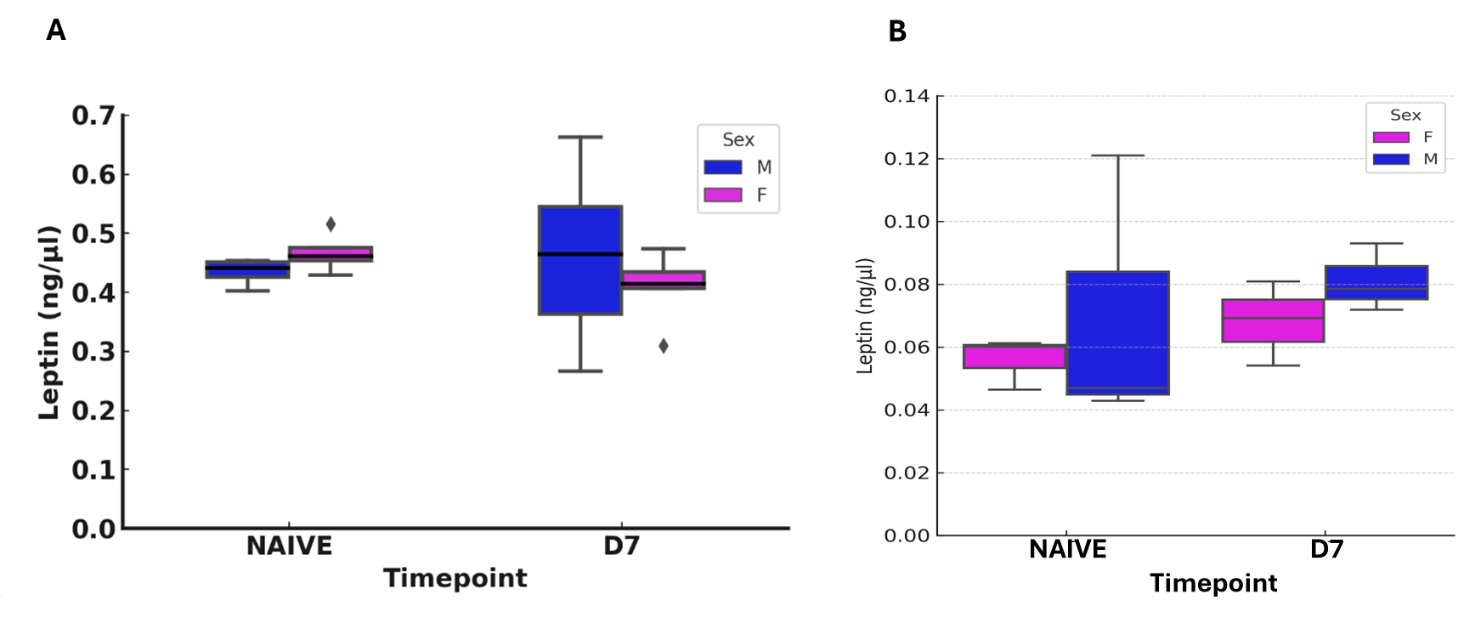


**Figure S3.** **Leptin levels in serum and subcutaneous adipose tissue (AT) of aged male and female mice before (naïve) and after sciatic nerve injury (CCI D7).** (A) Serum leptin concentration measured by ELISA, expressed as ng/μl. No significant effects of Sex (*p* = 0.47), Time (*p* = 0.39), or Sex × Time interaction (*p* = 0.94) were detected (two-way ANOVA).
(B) AT leptin concentration measured by ELISA, expressed as ng/μl. No significant effects of Sex (*p* = 0.35), Time (*p* = 0.43), or Sex × Time interaction (*p* = 0.96) were detected (two-way ANOVA). Data are shown as boxplots (blue = males; fuchsia = females).


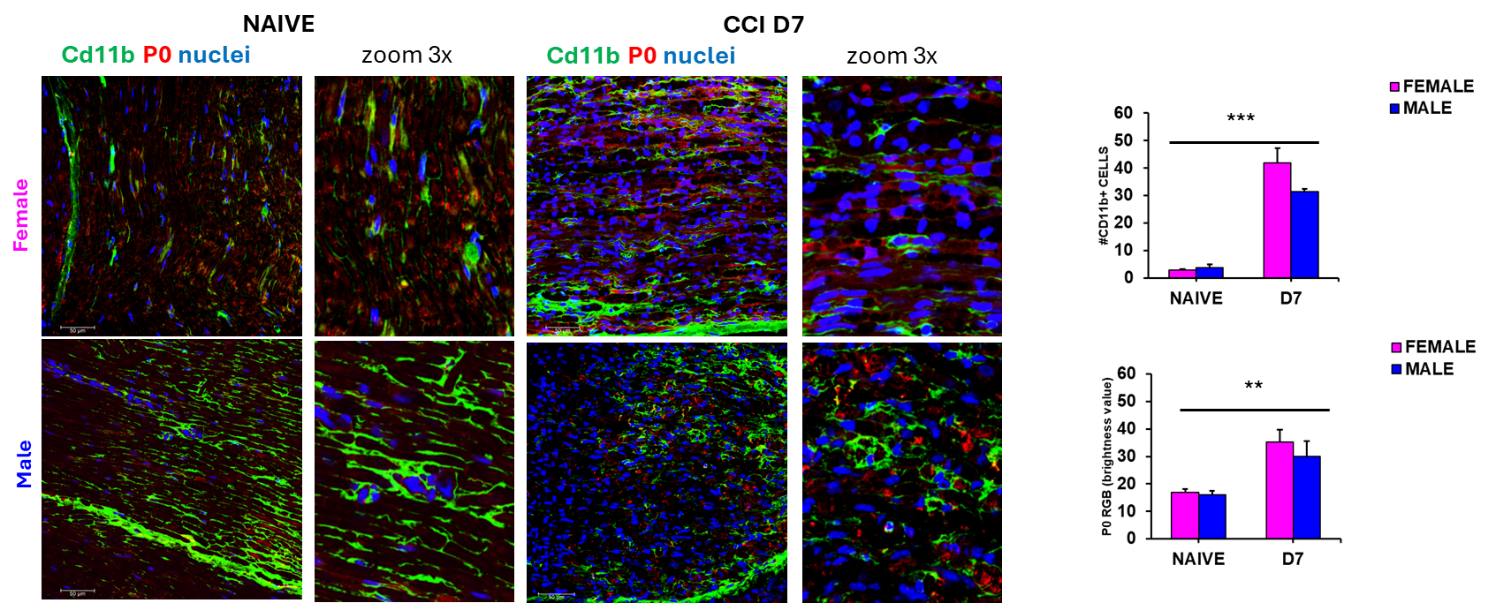


**Figure S4. Macrophage recruitment and P0 myelin protein changes in the injured sciatic nerve of aged male and female mice**. Representative immunofluorescence images of transverse sciatic nerve sections from naïve and CCI day 7 (D7) mice stained for CD11b (green), P0 (red), and nuclei (blue). Magnification 40x and 3× zoom images are shown to highlight cellular details. Scale bar = 50 μm. Quantification (right) shows:(A) Number of CD11b⁺ cells and (B) P0 fluorescence intensity (RGB brightness value). (N = 3 animals/group; 2 slices/animal). A significant increase in CD11b⁺ macrophages was detected at D7 compared to naïve in both sexes (ANOVA one-way F₃,₂₀ = 26.075, p < 0.0001; Tukey-Kramer: female D7 vs naïve p < 0.0001; male D7 vs naïve p < 0.0001). P0 fluorescence intensity also increased at D7 in both sexes (ANOVA one-way F₃,₂₀ = 6.45, p = 0.0031; Tukey-Kramer: female D7 vs naïve p < 0.005; male D7 vs naïve p < 0.005). The post-injury increase in P0 fluorescence likely reflects myelin aggregation during degeneration rather than increased protein synthesis, as reported in previous studies (16, 18, 19). This apparent increase reflects the aggregation of P0⁺ myelin profiles during degeneration, rather than an actual increase in protein content, as P0 levels typically decrease after injury.
